# Acute lymphoblastic leukemia-derived extracellular vesicles affect quiescence of hematopoietic stem and progenitor cells

**DOI:** 10.1038/s41419-022-04761-5

**Published:** 2022-04-12

**Authors:** Aleksandra Georgievski, Anaïs Michel, Charles Thomas, Zandile Mlamla, Jean-Paul Pais de Barros, Stéphanie Lemaire-Ewing, Carmen Garrido, Ronan Quéré

**Affiliations:** 1 UMR1231, Inserm/Université Bourgogne Franche-Comté, Dijon, France; 2 LipSTIC Labex, Dijon, France; 3 Plateforme de Lipidomique Analytique, Université Bourgogne Franche-Comté, Dijon, France; 4 Laboratoire de Biochimie Spécialisée, Hôpital Universitaire François Mitterrand, Dijon, France; 5 Centre Georges François Leclerc-Unicancer, Dijon, France

**Keywords:** Acute lymphocytic leukaemia, Cancer models, Cancer metabolism

## Abstract

Patient-derived xenografted (PDX) models were generated through the transplantation of primary acute lymphoblastic leukemia (ALL) cells into immunodeficient NSG mice. We observed that ALL cells from mouse bone marrow (BM) produced extracellular vesicles (EVs) with specific expression of inducible heat shock protein HSP70, which is commonly activated in cancer cells. Taking advantage of this specific expression, we designed a strategy to generate fluorescent HSP70-labeled ALL EVs and monitor the impact of these EVs on endogenous murine BM cells ex vivo and in vivo. We discovered that hematopoietic stem and progenitor cells (HSPC) were mainly targeted by ALL EVs, affecting their quiescence and maintenance in the murine BM environment. Investigations revealed that ALL EVs were enriched in cholesterol and other metabolites that contribute to promote the mitochondrial function in targeted HSPC. Furthermore, using CD34^+^ cells isolated from cord blood, we confirmed that ALL EVs can modify quiescence of human HSPC. In conclusion, we have discovered a new oncogenic mechanism illustrating how EVs produced by proliferative ALL cells can target and compromise a healthy hematopoiesis system during leukemia development.

## Introduction

In mice models, leukemia cells create bone marrow (BM) niches that disrupt the behavior of normal hematopoietic stem cells (HSC) [1–4]. Recent studies have described how tumor-derived extracellular vesicles (EVs) played a crucial role in manipulating the tumor microenvironment to benefit the cancer cells [5–8]. EVs secreted by leukemia cells can transport cargoes (proteins, RNAs, lipids) from cancer cells to BM stromal cells and therefore contribute to the formation of a leukemia-supportive BM microenvironment [9, 10]. Acute myeloid leukemia (AML)-derived EVs were found to induce IL-8 production in BM stromal cells, protecting the leukemia cells against chemotherapy [11]. IL-8 secreted from BM stromal cells treated with chronic myelogenous leukemia (CML) EVs bound to two chemokine receptors and increased cell adhesion, motility, and survival of CML cells [12]. The alteration of the BM-mesenchymal stem cells (MSC) by EVs released by CML cells has been furthermore characterized [13]. CML EVs induced the transition of stromal cells into cancer-associated fibroblasts with enhanced proliferation, migration, and secretion of inflammatory cytokines, contributing to a tumor-supportive microenvironment [14]. The interaction of CML EVs with stromal cells also allowed leukemia cells to adhere more to stromal cells [15]. Furthermore, EVs released from leukemia cells were responsible for the suppression of HSC function by two major mechanisms, indirectly through the stromal reprogramming of niche-retention factors or as a consequence of leukemia EVs-directed carrier delivery to HSC [10, 16–20].

Tumor cells have to recondition their metabolism, so they require chaperones for their survival, particularly the 70 kDa heat shock protein (HSP70). Expression of the *Heat shock 70* *kDa protein 1* (*HSPA1A*) gene is inducible and was associated with poor prognosis in an extensive number of cancers, while involved in tumor growth, invasion, migration and resistance to anti-cancer therapy [21–28]. Previous studies in CML [29] and AML [30, 31] suggested that HSP70 is also overexpressed in hematological malignancies.

Acute lymphoblastic leukemia (ALL)-derived EVs have been poorly investigated so far [32–34]. In this study, using patient-derived xenografted (PDX) models to study T-ALL and B-ALL [35–37], we discovered that ALL cells in mouse BM produced EVs with a specific expression of HSP70. Through the use of fluorescent HSP70-labeled ALL EVs, we monitored the impact of these EVs on endogenous BM cells. We showed that the main target of these ALL EVs were HSPC, whose maintenance was affected. While ALL EVs were produced by highly proliferative leukemia cells, the exhaustion of the healthy HSPC was related to the high levels of lipids and metabolites in ALL EVs. Their transfer to HSPC contributed to promote mitochondrial activity and compromise the quiescence and homeostasis of healthy HSPC.

## Results

### T-ALL and B-ALL cells express high levels of HSP70 in PDX mice

PDX mice were sacrificed at day 35 post transplantation, when >80% of ALL cells were detected in BM (Fig. S1). We conjugated the HSP70 peptide aptamer that allowed the detection of murine and human HSP70 protein [26] with the ATTO488 fluorescent label and performed immunofluorescence on BM sections of ALL PDX mice. We noticed a strong HSP70 expression in ALL cells, while the expression was low in mouse microenvironment (Fig. S2A). Bones were crushed to recover BM cells and by flow cytometry, human ALL cells expressed marked levels of intracellular HSP70, compared with healthy mouse BM cells (Fig. S2B). Ki67 staining showed that cells in S and G2/M phases displayed a significant expression of HSP70 (*P* < 0.0001, Fig. S2C). Observation of HSP70 staining using fluorescent microscopy after fluorescent-activated cell sorting (FACS) depicted a high expression in the cytoplasm of dividing cells (Fig. S2D). In conclusion, HSP70, which is known to be involved in tumor growth [22–27] was highly expressed by ALL cells in PDX models.

### ALL cells produce their own EVs in the BM of PDX mice

**Fig. 1:**
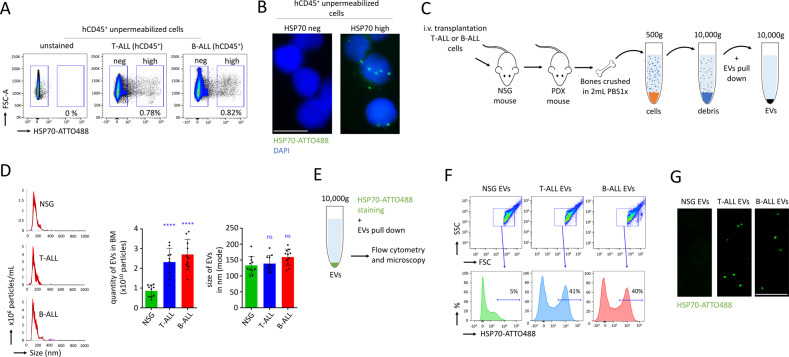
T-ALL and B-ALL PDX mice produced EVs with membrane-anchored HSP70 expression. **A** Flow cytometry showing that few ALL cells (<1%) were expressing high level of HSP70. Immunostaining for HSP70 expression performed without permeabilization on BM cells from T-ALL and B-ALL PDX models. Flow cytometry is shown on hCD45^+^ cells for T-ALL and B-ALL viable cells (FVS450 negative). **B** Immunostaining on cells without permeabilization. Cells expressing HSP70 (HSP70 high) or negative for expression (HSP70 neg) were purified by FACS and observed by microscopy. EVs with membrane-anchored HSP70 expression were observed among the population of cells expressing HSP70. Example of microscopy for B-ALL cells. Microscopy with magnification ×63, scale bar represents 10 µm. **C** Procedure followed to isolate EVs in BM of PDX mice developing ALL. **D** Quantification and size of EVs isolated from BM with the NTA. Data are shown as means ± SD; *n* = 9 mice. *P* value measured by one-way Anova with Tukey’s multiple comparison test; ****P < 0.0001; ns, non-significant. **E** Procedure followed to isolate stain EVs prior to pull-down and analysis. **F** Flow cytometry confirming expression of HSP70 by EVs produced in T-ALL and B-ALL PDX mice, compared with control EVs produced by an NSG mouse. Data are representative of two independent experiments. **G** Microscopy on EVs purified from control NSG and ALL PDX models showing fluorescent EVs stained with HSP70-ATTO488, microscopy with magnification ×63, scale bar represents 5 µm.

In tumor cells, HSP70 can be found anchored in the plasma membrane [24, 38] and tumor-derived EVs express membrane HSP70, which have been involved in tumor growth [25, 27, 28]. In this study, we observed that a small population (<0.1%) of cells were showing cell surface expression of HSP70 in unpermeabilized ALL cells freshly isolated from the BM (Fig. 1A). Cells expressing a high level of HSP70 were analyzed after FACS by microscopy, and fluorescent EVs were detected (Fig. 1B). Ki67 staining furthermore suggested that EVs were produced independently of cell cycle activity (Fig. S2E). Next, we developed a protocol to pull-down the EVs by successive centrifugations of BM cells’ supernatants (Fig. 1C). Nanoparticle tracking analysis (NTA) showed increased production of EVs from the BM of PDX mice, compared with control NSG (*P* < 0.0001, Fig. 1D). When we analyzed the size of the EVs by NTA, we found no significant differences (100–150 nm, Fig. 1D). Transmission electron microscopy (TEM) depicted that the size of the EVs corresponded more to large EVs (also called microvesicles or ectosomes) than to small EVs (such as exosomes) (Fig. S3A). Through mass spectrometry, we detected human and murine proteins isolated from EVs purified from the BM of PDX mice, which confirmed the release of EVs from both murine healthy endogenous BM cells and human neoplastic ALL cells. We focused our study on human proteins that were commonly expressed in T-ALL and B-ALL EVs (391 proteins), and compared their level of expression to the endogenous murine proteins found in control NSG EVs. Approximately 10% of the proteins (43 proteins) were found to be specific to ALL EVs, including inducible HSP70 (HSPA1A) (Fig. S3B-G). The proteomic analysis further identified several specific markers for large EVs (Table S1). This confirmed that the observed EVs belong to the group of large size EVs, also called ectosomes. Since only EVs isolated from ALL PDX mice showed specific detection of HSP70, we developed a protocol to stain EVs with the HSP70 peptide aptamer conjugated with the ATTO488 fluorescent dye, just before EVs were pulled-down (Fig. 1E). The composition of the EVs was then characterized by flow cytometry: in T-ALL and B-ALL EVs, ~40% of particles showed expression of HSP70 while no expression was detected in control NSG EVs (Fig. 1F). These findings were then confirmed by fluorescent microscopy (Fig. 1G). In conclusion, we were able to track EVs produced by T-ALL and B-ALL cells in the BM of PDX mice using membrane-anchored HSP70 expression.

### Murine HSPC intake of ALL cells’ EVs

**Fig. 2:**
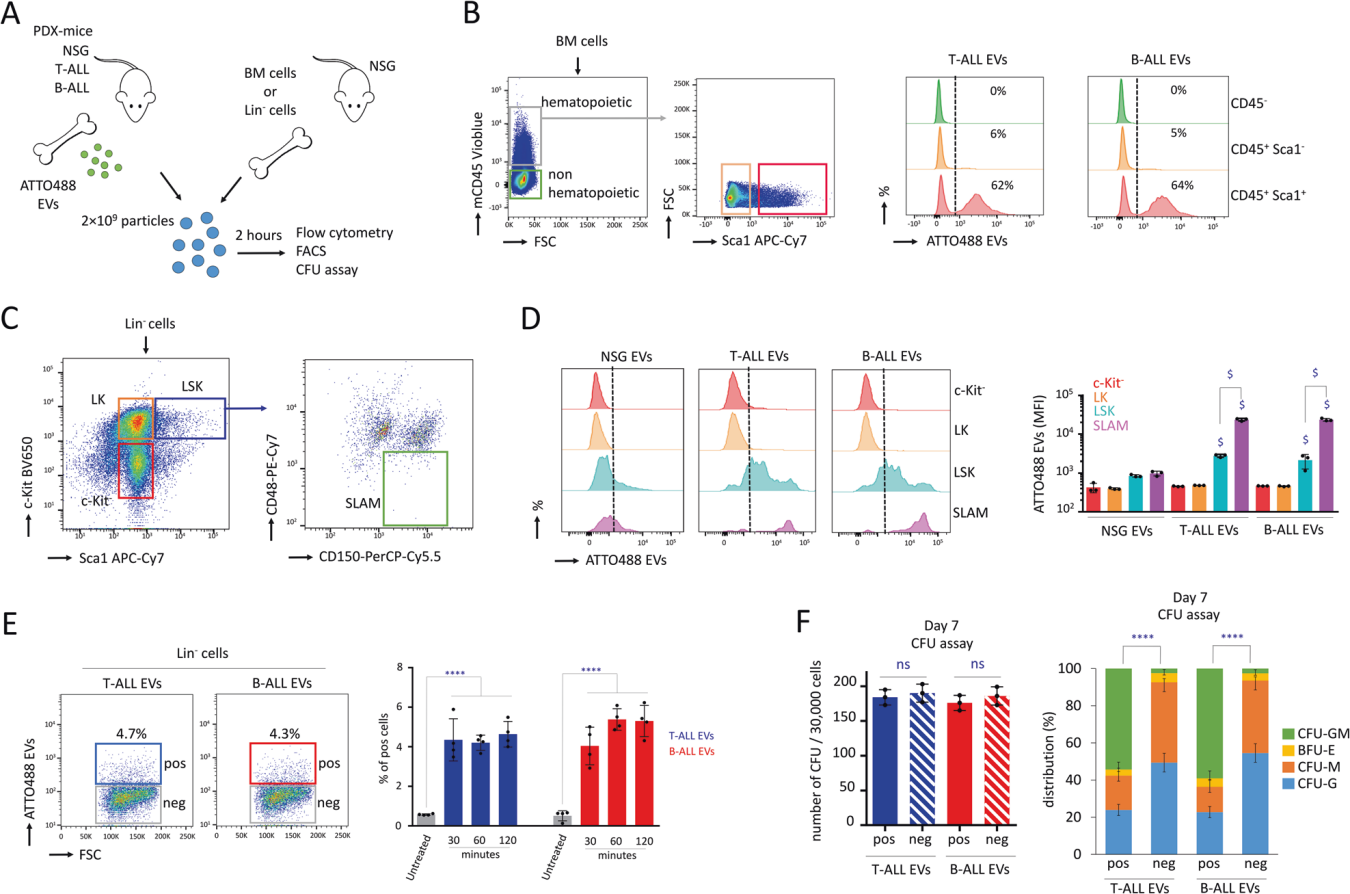
Murine HSPC intake of T-ALL and B-ALL EVs. **A** Procedure followed to identify populations of murine cells that take in fluorescent ALL EVs. **B** Flow cytometry on total BM cells demonstrating that primitive hematopoietic cells (Sca1^+^ mCD45^+^) take in fluorescent T-ALL and B-ALL EVs (ATTO488 EVs). **C** Flow cytometry gating strategy used to analyze further subpopulations among the primitive Lin^−^ cells and their capacity to take in ALL EVs. **D** Mean fluorescence intensity (MFI) on Lin^−^ cells showing that HSPC (LSK; Lin^−^ Sca1^+^ c-Kit^+^) and HSC (SLAM; LSK CD150^+^ CD48^−^) can take in ALL EVs (ATTO488 EVs), while LK (Lin^−^ c-Kit^+^ Sca1^−^) cells and the c-Kit negative (c-Kit^−^) cells cannot. The intake of SLAM cells was higher than LSK cells. Data are compared to c-Kit^−^ cells and are shown as means ± SD; *n* = 3 mice. *P* value measured by one-way Anova with Tukey’s multiple comparison test; ^$^*P* < 0.0001. **E** Among Lin^−^ cells, cells showing intake of ALL EVs (ATTO488 EVs positive cells; pos) and no intake (ATTO488 EVs negative cells; neg). To examine the cell intake of EVs, Lin^−^ cells are treated with HSP70-ATTO488 EVs, for 30 min to 2 h. Data showing that ALL EVs have the potential to rapidly bind, within 30 min, ~4% of the Lin^−^ cells. Data are shown as means ± SD; *n* = 4 biological replicates. *P* value measured by one-way Anova with Tukey’s multiple comparison test; ^****^P < 0.0001. **F** CFU assay is assessed for 30,000 pos and neg cells purified by FACS, after 7 days on semi-solid media. Data showing that pos cells are more primitive, while they produce more CFU-GM colonies when compared with neg cells. Quantity of CFU (left panel) and distribution (right panel). Data are shown as means ± SD; *n* = 3 mice. *P* value measured by two-tailed unpaired Student’s *t* test; ^****^P < 0.0001; ns, non-significant.

After confirming that T-ALL and B-ALL cells produced EVs in BM with membrane-anchored HSP70, we used fluorescent EVs to track the modality of the intake of ALL EVs in murine BM cells. We incubated HSP70-ATTO488-positive EVs with total murine BM cells in vitro for two hours, to identify population of cells that was targeted by ALL EVs (Fig. 2A). By flow cytometry, in the ATTO488 channel, we observed that ~60% of the primitive hematopoietic cells (mCD45^+^ Sca1^+^) were positive, while the non-primitive hematopoietic cells and the non-hematopoietic cells were negative (Fig. 2B). Then, we analyzed different subpopulations among the lineage negative (Lin^−^) cells (Fig. 2C). We observed that HSPC (LSK; Lin^−^ Sca1^+^ c-Kit^+^) and HSC (SLAM; LSK CD150^+^ CD48^−^) were able to take in the fluorescent EVs, while no intake was detected for late progenitors (LK; Lin^−^ Sca1^−^ c-Kit^+^) or c-Kit^−^ cells (Fig. 2D). We next treated Lin^−^ cells with HSP70-ATTO488 EVs, from 30 min to 2 h and observed that ALL EVs rapidly bound, within 30 min, ~4% of the Lin^−^ cells (Fig. 2E). After FACS of ATTO488 positive Lin^−^ cells, no difference was observed regarding the number of colony-forming units (CFU) following 7 days of culture on semi-solid media (*P* > 0.05), which suggested that the intake of EVs did not alter cell viability. Interestingly, the CFU distribution provided evidence that cells showing intake of ALL EVs were more primitive, since they produced more CFU-GM colonies (*P* < 0.0001, Fig. 2F). In conclusion, only the primitive murine HSPC in BM were able to uptake the EVs from ALL cells.

### The intake of ALL EVs by HSPC is mediated by lipid raft endocytosis

**Fig. 3:**
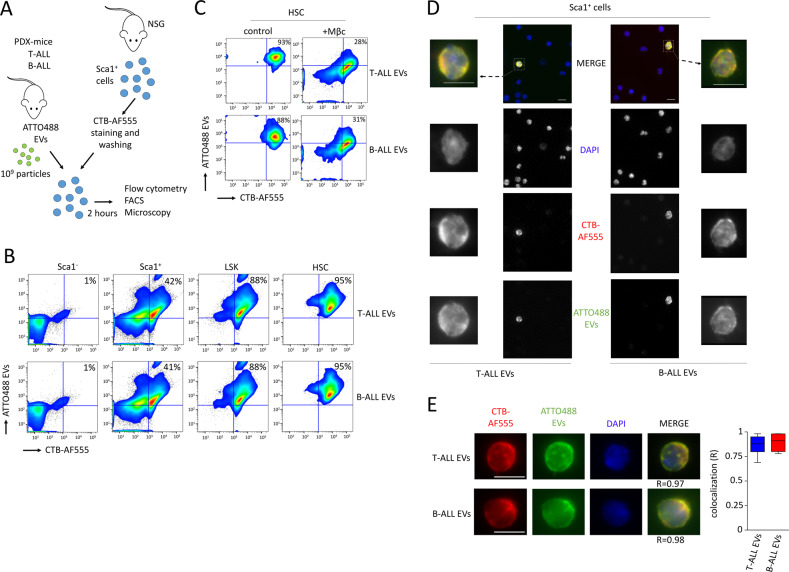
Intake of T-ALL and B-ALL EVs by lipid raft-enriched HSPC. **A** Procedure followed to identify populations of murine Lin^−^ cells that take in fluorescent ALL EVs. Murine Sca1^+^ cells were purified with magnetic beads and stained with CTB prior to incubation with fluorescent ALL EVs (HSP70-ATTO488). **B** Flow cytometry on different populations showing that LSK and HSC (SLAM cells) express high levels of lipid rafts (CTB-AF555) as well as intake of ALL EVs (ATTO488 EVs). Sca1^+^ cells show intermediate levels in expression of lipid rafts and ALL EV intake, while Sca1^−^ cells are not positive for lipid rafts expression or for intake of ALL EVs. Representative of two independent experiments. **C** Flow cytometry on HSC showing that when Sca1^+^ cells are pretreated for 2 h with Methyl-β-cyclodextrine (Mβc), this affects the lipid rafts staining (CTB-AF555) as well as the intake of ALL EVs (ATTO488 EVs). **D** Microscopy on murine Sca1^+^ cells showing that the population of primitive hematopoietic cells which take in fluorescent EVs (ATTO488 EVs) correspond to cells also displaying lipid raft staining with the cholera toxin subunit B (CTB-AF555). Magnification ×40, scale bar represents 5 µm. Representative of two independent experiments. **E** Single cell sorting on positive cells showing colocalization score (R) between EV intake and lipid rafts. Data shows box-and-whisker plots (*n* = 30 cells for each conditions), presented as medians (central line), first and third quartiles (bottom and top of boxes, respectively), and whiskers (extreme values). Fluorescent optical sections of cells, magnification ×63, scale bar represents 5 µm.

We generated artificial lipid vesicles containing HSP70, which failed to bind HSPC in vitro. Therefore, interaction of ALL EVs with HSPC was not mediated via membrane bound-HSP70 (Fig. S4). Further mechanisms can explain the intake of EVs, including the lipid rafts mediating endocytosis [39, 40]. As platforms for membrane trafficking and signal transduction, lipid rafts were found highly expressed by HSPC [41–46]. We therefore stained Sca1^+^ cells with cholera toxin subunit B (CTB) to detect the lipid rafts, washed cells and monitored cell intake of EVs over the following 2 h in vitro (Fig. 3A). Using flow cytometry, lipid rafts were not detected on Sca1^−^ cells, and this population showed no uptake of ALL EVs. Among Sca1^+^ cells, ~40% of the cells showed lipid raft staining and intake of ALL EVs. HSPC (LSK cells) showed high detection of lipid rafts and relevant intake of ALL EVs (~88% of cells). Finally, HSC (SLAM cells), which took up the most ALL EVs, also showed the most elevated staining of lipid rafts (~95% of cells) (Fig. 3B). Lipid rafts are cholesterol-enriched patches located in the plasma membrane, and the dynamic protein assembly in these lipid rafts can be modified by a disturbance in the lipid composition following methyl-β-cyclodextrine treatment, a cholesterol-removing agent used for lipid raft disruption [41]. Treatment with this chemical compound affected the intake of fluorescent EVs by HSC (Fig. 3C). Thus, murine BM cells expressing high levels of lipid rafts such as HSPC and HSC can intake ALL EVs and the use of a drug inducing lipid raft disturbance reduced the intake of fluorescent ALL EVs, which demonstrated that EVs intake by HSC was mediated by lipid raft-dependent endocytosis. By microscopy, Sca1^+^ cells with marked expression of lipid rafts were also the ones showing important intake of the fluorescent EVs (Fig. 3D). Single cell microscopy confirmed furthermore the relevant colocalization between lipid rafts and the intake of the EVs (Fig. 3E).

### EVs produced by PDX mice affect maintenance of murine HSC in vivo

**Fig. 4:**
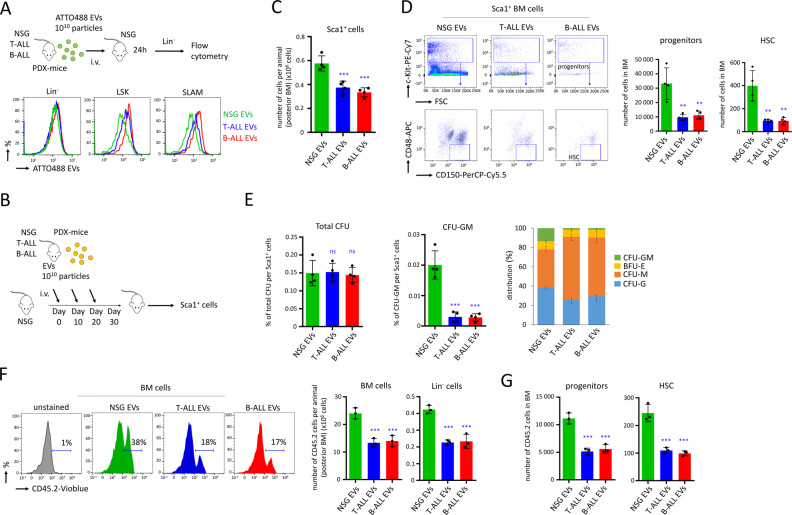
Injection of ALL EVs in vivo affects maintenance of murine HSC. **A** NSG mice were injected with fluorescent EVs (ATTO488 EVs) by i.v. injections at 10^10^ particles/mouse. Mice were sacrificed 24 h after the injection, and florescence was determined by flow cytometry on Lin^−^, LSK (c-Kit^+^ Sca1^+^) and SLAM (LSK CD150^+^ CD48^−^) cells. **B** Procedure followed to treat NSG mice with ALL EVs or control EVs, through three consecutive i.v. injections, at 10^10^ particles/mouse/every 10 days. Ten days after the third injection, mice were sacrificed to perform experiments on Sca1^+^ cells. **C** The absolute number of Sca1^+^ cells detected in BM. Data are shown as means ± SD; *n* = 4 mice. **D** The number of hematopoietic progenitors (CD45^+^ Sca1^+^ c-Kit^+^ cells) and HSC (SLAM; LSK CD150^+^ CD48^−^ cells) were determined by flow cytometry. Data are shown as means ± SD; *n* = 4 mice. **E** Sca1^+^ cells (10^5^ cells) isolated from mice were analyzed on semi-solid methylcellulose media for growth of murine hematopoietic CFU. Data show the percentage of total CFU and CFU-GM per Sca1^+^ cells, as well as the distribution among colonies. Data are shown as means ± SD; *n* = 4 mice. **F** Sca1^+^ cells (3 × 10^5^ cells) were injected in C57BL/6.SJL (Ly.1) mice and the hematopoietic reconstitution was examined by flow cytometry, 4 weeks after the transplantation. Absolute number of CD45.2 total and Lin^−^ BM cells. Data are shown as means ± SD; *n* = 3 transplanted mice. **G** Hematopoietic reconstitution for progenitors (LSK cells) and HSC (SLAM cells). Absolute number of CD45.2 cells in BM. Data are shown as means ± SD; *n* = 3 transplanted mice. On this figure, *P* value is measured by one-way Anova with Tukey’s multiple comparison test; **P < 0.01; ***P < 0.001; ns, non-significant.

Through the i.v. injection of ALL fluorescent EVs in vivo, we provided evidence that, within 24 h, T-ALL EVs and B-ALL EVs targeted HSPC and HSC (Fig. 4A). We consequently performed experiments to confirm the role of EVs produced by ALL cells on the maintenance of murine HSC in vivo, following three consecutive i.v. injections (10^10^ particles/mouse, Fig. 4B). We detected a reduction of the absolute number of Sca1^+^ cells (*P* < 0.001, Fig. 4C), but neither a reduction of the total number of BM cells nor Lin^−^ cells (Fig. S5). By flow cytometry, we also observed among the Sca1^+^ BM cells a reduction of the absolute number of progenitors (*P* < 0.01, c-Kit^+^ Sca1^+^ cells) and HSC (*P* < 0.01, c-Kit^+^ Sca1^+^ CD150^+^ CD48^−^) (Fig. 4D). When Sca1^+^ cells, isolated from NSG mice injected with T-ALL EVs or B-ALL EVs, were cultured on semi-solid media, a considerable decrease of the percentage of CFU-GM per Sca1^+^ cells was detected (*P* < 0.001, Fig. 4E), suggesting exhaustion of the most primitive HSPC. The subsequent analysis of the reconstitution capacity in vivo was performed following the transplantation of Sca1^+^ cells in recipient C57BL/6.SJL (Ly.1) mice. The capacity of Sca1^+^ cells, isolated from NSG mice treated with ALL EVs to reconstitute hematopoiesis, was reduced by twofold, 4 weeks post-transplantation in recipient mice, as assessed by the absolute number of CD45.2 total and Lin^−^ cells in BM (*P* < 0.001, Fig. 4F). Further, we observed a reduction among the quantity of reconstituted progenitors (LSK cells) and HSC (SLAM cells) in BM (*P* < 0.001, Fig. 4G). In conclusion, when EVs produced in the BM by ALL PDX mice were injected in vivo, an attrition of the murine HSPC occurred following 4 weeks. We can therefore conclude that the maintenance of HSPC was affected precisely by EVs produced by ALL cells in BM.

### ALL EVs do not affect ALL development in PDX mice

We assessed the effect of EVs on MS5 stromal murine cells in vitro. Using flow cytometry and microscopy, the intake of fluorescent EVs was confirmed (Fig. S6A–B). Three days after the treatment, we observed no activation of further signaling pathways (Fig. S6C) and wound healing assay established that there was no acceleration in the growth of MS5 cells after exposure to ALL EVs (Fig. S6D). Treatment with ALL EVs did not modify also the ability of the leukemia cells or normal primitive hematopoietic cells to adhere to stromal MS5 cells in vitro (Fig. S7A–B). We furthermore observed that the pre-conditioning of mice with i.v. injection of ALL EVs did not accelerate the development of T-ALL or B-ALL in vivo (Fig. S8). These results allowed us to conclude that EVs released by ALL cells did not contribute to the formation of a leukemia-supportive BM microenvironment.

### Human ALL development in PDX mice induces exhaustion of HSPC

**Fig. 5:**
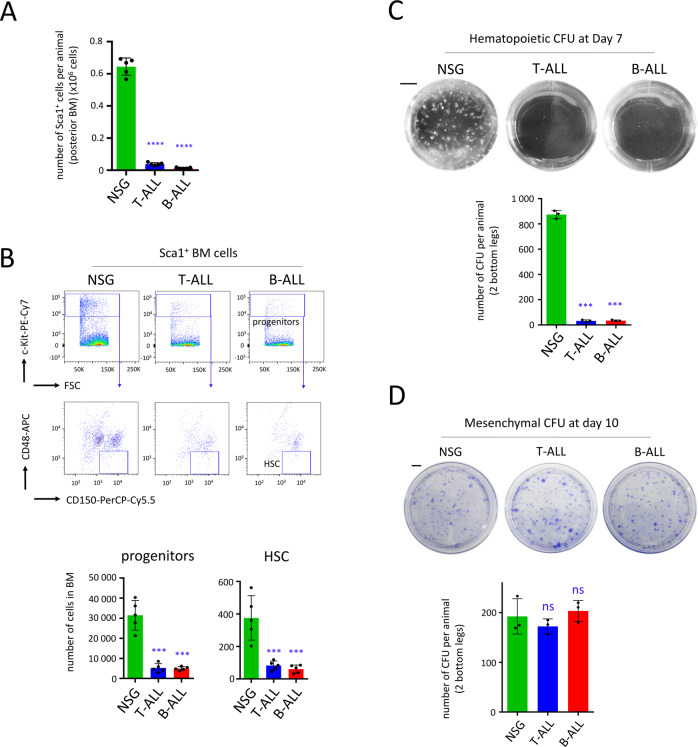
Exhaustion of HSPC but not MSC in ALL PDX mice. Mice were injected with 5 × 10^5^ cells (T-ALL) or 10^5^ cells (B-ALL) and murine Sca1^+^ cells were recovered when mice were developing ALL disease (day 35). **A** The number of Sca1^+^ cells recovered from the BM of control NSG mice and PDX mice developing T-ALL or B-ALL. Data are shown as means ± SD; *n* = 5 mice. **B** Analysis of Sca1^+^ cells by flow cytometry showing loss of hematopoietic progenitors (CD45^+^ c-Kit^+^) and HSC (SLAM; CD150^+^ CD48^−^) in T-ALL and B-ALL PDX mice. Data are shown as means ± SD; *n* = 5 mice. **C** 25% of the Sca1^+^ cells were cultured in methylcellulose media and hematopoietic CFU were observed at day 7; scale bar represents 5 mm. Data are shown as means ± SD; *n* = 3 mice. **D** 75% of the Sca1^+^ cells were cultured in media for growth of mesenchymal CFU for 10 days, scale bar represents 1 cm. Data are shown as means ± SD; *n* = 3 mice. On this figure, *P* value is measured by one-way Anova with Tukey’s multiple comparison test; ***P < 0.001; ****P < 0.0001; ns non-significant.

Next, we assessed the effect of leukemia development in our PDX models, and observed a major exhaustion of murine Sca1^+^ cells (*P* < 0.0001) in the BM of mice developing T-ALL and B-ALL (Fig. 5A). By flow cytometry, we detected a considerable reduction of progenitors as well as HSC (*P* < 0.001, Fig. 5B). We cultured Sca1^+^ cells in specific media to support growth of either hematopoietic or mesenchymal CFU and compared these samples with Sca1^+^ cells isolated from the BM of control NSG mice. We detected a considerable decrease of hematopoietic CFU (*P* < 0.001), when Sca1^+^ cells isolated from PDX models were cultured in semi-solid media (Fig. 5C), while the quantity of mesenchymal CFU remained unchanged (*P* > 0.05, Fig. 5D). Using a proteomic array, we analyzed more than one hundred murine cytokines, chemokines and growth factors isolated from the BM environment of PDX mice. While most of the secreted proteins showed no alteration in their expression rates, three murine proteins were dysregulated (Igfbp2, Ccl21 and Mmp9, Fig. S9). However, any of these dysregulations could explain the attrition of normal HSPC observed in ALL PDX mice [47–49].

### ALL EVs affect the quiescence of murine HSPC ex vivo

**Fig. 6:**
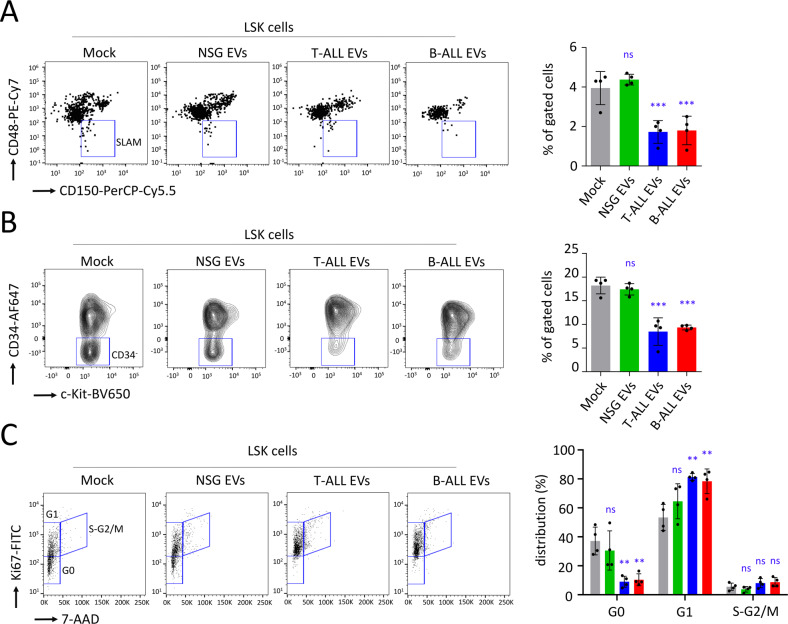
T-ALL and B-ALL EVs affect HSPC maintenance in vitro. Murine Lin^−^ cells were treated ex vivo with EVs isolated from NSG control mice or ALL PDX models. **A** After 24 h, as assessed by flow cytometry on LSK gating cells, treatment with ALL EVs affects the numbers of HSC (SLAM). **B** This is further corroborated by an attrition of the primitive LSK CD34^−^ cells. **C** Flow cytometry on LSK gating cells showing a loss of quiescent cells (in G0). On this figure, data are shown as means ± SD; *n* = 4 mice. *P* value are measured by one-way Anova with Tukey’s multiple comparison test; ^**^P < 0.01; ^***^P < 0.001; ns, non-significant.

We treated murine Lin^−^ cells with EVs ex vivo and assessed the effect on hematopoietic subpopulations after 24 h of exposure. No apoptosis was observed in HSPC and no impact was detected on the proportion of LSK cells, late progenitors (LK cells), or on the distribution of mature progenitors (Fig. S10A). The total amount of CFU at day 7, as well as the distribution between the different types of colonies, were similar for all conditions (Fig. S10B). We could not detect any effect on further signaling pathways in LSK cells, following 24 h of exposure to ALL EVs, including the TGFβ signaling involved in the maintenance of quiescent HSC [45, 50–52] (Fig. S10C–D). Nevertheless, after exposure to ALL EVs for 24 h, we detected a marked attrition of HSC (*P* < 0.001, Fig. 6A) and in LSK CD34^−^ cells (*P* < 0.001, Fig. 6B). Using anti-Ki67 antibody staining and flow cytometry, we detected a significant decrease of G0 quiescent LSK cells (*P* < 0.01, Fig. 6C).

We treated human CD34^+^ HSC recovered from cord blood with EVs. Following 24 h of exposure, we observed a marked loss of quiescence, using Ki67 staining and flow cytometry (*P* < 0.01, Fig. S11A). CD34^+^ cells were i.v. injected into sub-lethally irradiated NSG recipient mice. Four weeks after the transplantation, the human hematopoiesis reconstitution assessed by flow cytometry in BM was found tenfold lower, when mice were injected with CD34^+^ cells exposed to T-ALL or B-ALL EVs, compared with mice transplanted with CD34^+^ cells either untreated or exposed to NSG EVs (Fig. S11B). Then, ALL EVs affected the self-renewal activity of cord blood CD34^+^ cells. In conclusion, EVs isolated from ALL PDX mice affected the quiescence of murine HSC, as well as human CD34^+^ cord blood HSC.

### ALL EVs are highly enriched with cholesterol

**Fig. 7:**
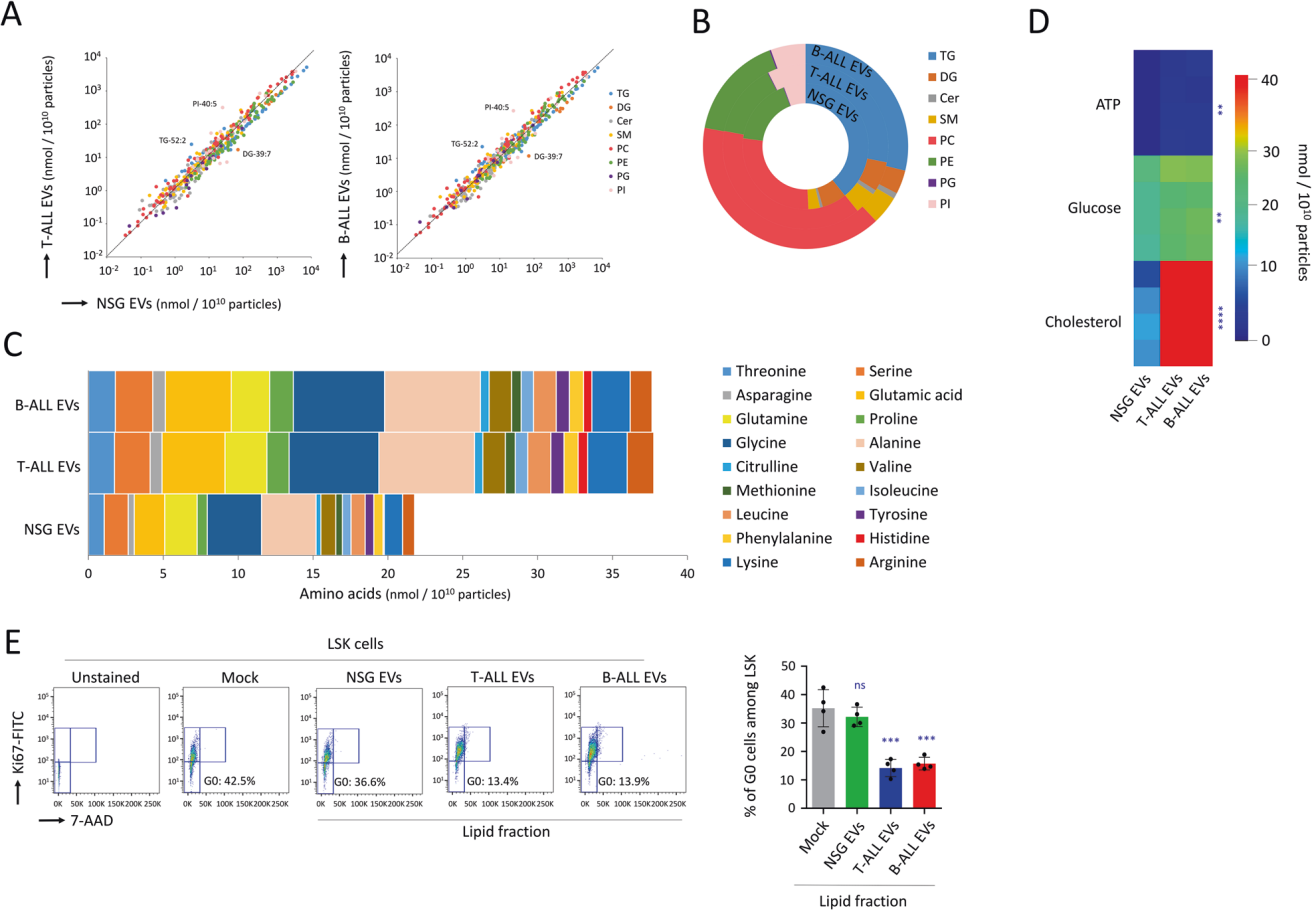
T-ALL and B-ALL EVs are enriched with metabolites and cholesterol. **A** UPLC-LC-MS/MS data showing the quantity of all lipids (nmol/10^10^ particles) identified in T-ALL EVs and B-ALL EVs compared with NSG EVs. Triacylglycerides (TG), diacylglycerides (DG), ceramides (Cer), sphingomyelins (SM), phospho-cholines (PC), phospho-ethanolamines (PE), phospho-glycerols (PG), phospho-inositols (PI). **B** Distribution of each family of lipids in T-ALL EVs, B-ALL EVs and NSG EVs. **C** Data showing increased quantities of other amino acids measured by UPLC LC-MS/MS in T-ALL EVs and B-ALL EVs when compared with NSG EVs. **D** Data showing how cholesterol was highly present in T-ALL EVs and B-ALL EVs when compared with NSG EVs. ALL EVs also show elevated levels of ATP and glucose; *n* = 4 mice. *P* value measured by one-way Anova with Tukey’s multiple comparison test; **P < 0.01; ****P < 0.0001. **E** Flow cytometry data showing a relevant loss of quiescent cells (G0) with the lipid fractions isolated from T-ALL or B-ALL EVs when compared with lipids isolated from control NSG mice. Data are shown as means ± SD; *n* = 4 mice. *P* value measured by one-way Anova with Tukey’s multiple comparison test; ***P < 0.001; ns, non-significant.

Following MitoTracker staining and flow cytometry, we detected the same quantity of mitochondria in ALL cells and in the endogenous mouse BM cells (Fig. S12A). However, tetramethylrhodamine (TMRM) staining revealed a higher mitochondrial potential followed in ALL cells (*P* < 0.0001, Fig. S12B), suggesting that ALL cells were highly metabolically active in vivo. The molecular composition of EVs reflected the features of the parent cell, and this included also metabolites and bioactive lipids [53–55]. Lipidomic analysis on EVs showed that, the quantity of lipids in ALL EVs and in NSG EVs were essentially the same (Fig. 7A). However, there was an increase within the total composition for phosphatidyl-cholines (PC) and sphingomyelins (SM), and a decrease for triacylglycerides (TG) (Fig. 7B). We also observed a higher quantity of all amino acids in ALL EVs compared with NSG EVs (Fig. 7C). We also detected higher levels of ATP and glucose in ALL EVs (*P* < 0.01), and cholesterol was interestingly the compound that was the most increased (*P* < 0.0001, Fig. 7D). Cholesterol is a polar molecule that cannot cross the plasma membrane, and only low-density lipoprotein (LDL) was considered to transfer cholesterol to HSC [56, 57]. Among several metabolites detected in ALL EVs tested individually, only LDL induced a relevant exhaustion of quiescent HSPC (*P* < 0.0001, Fig. S13A), and, following TMRM staining and flow cytometry, we established that LDL activated the mitochondrial membrane potential in LSK cells (*P* < 0.01, Fig. S13B). The important role of cholesterol was further corroborated when the lipid fraction isolated from ALL EVs using Folch’s method induced a relevant decrease of HSPC quiescence (*P* < 0.001, Fig. 7E) as well as an activation of mitochondrial membrane potential (*P* < 0.001, Fig. S13C). In conclusion, EVs produced by ALL cells were remarquably enriched with cholesterol, and we established that this compound was mainly responsible for loss of HSPC quiescence.

### ALL EVs enhance the mitochondrial potential of HSPC

**Fig. 8:**
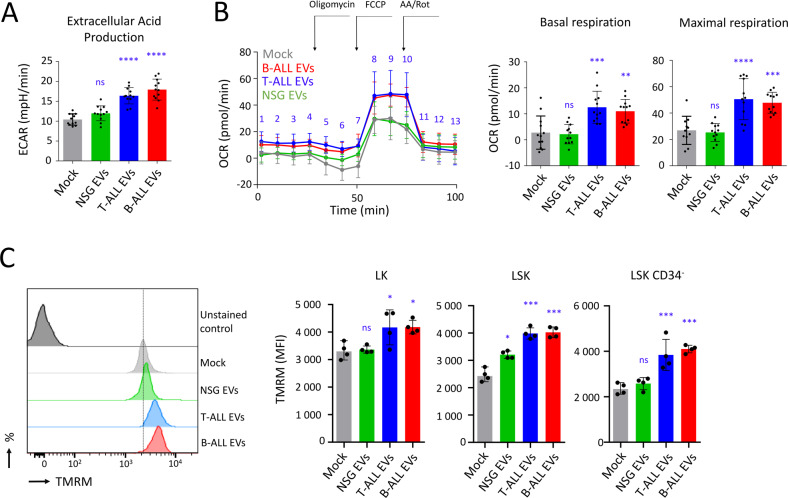
T-ALL and B-ALL EVs activate mitochondrial activity in HSPC. **A** Extracellular acid production measured with the Seahorse on Lin^−^ cells after 24 h of treatment with T-ALL EVs, B-ALL EVs, or NSG EVs (2 × 10^9^ particles); *n* = 4 mice, in technical triplicates. **B** Oxygen consumption rate (OCR) measured with the Seahorse on Lin^−^ cells after 24 h of treatment with T-ALL EVs and B-ALL EVs, compared with NSG EVs. Data showing that the basal and maximal respiration were increased following 24 h of Lin^−^ cell exposure to ALL EVs (2 × 10^9^ particles). Basal respiration corresponds to means of OCR measured for points 1–4 and maximal respiration for points 8–10. Oligomycin (1.5 μM), FCCP (1 μM), antimycin A, rotenone, (AA/Rot, 0.5 μM/0.5 μM); *n* = 4 mice, in technical triplicates. **C** Flow cytometry showing increased detection of the mitochondrial membrane potential states following the exposure of Lin^−^ cells to ALL EVs (2 × 10^9^ particles) for 24 h. Cells were treated with TMRM for 30 min before flow cytometry recording. Data performed on Lin^−^ cells and gating on LK, LSK and LSK CD34^−^ cells. Example of plot for TMRM recording with dashed line corresponding to mean fluorescence intensity (MFI) for Mock. On this figure, (**A**, **B**) data are shown as means ± SD; *n* = 4 mice in technical triplicates, (**C**) data are shown as means ± SD; *n* = 4 mice. *P* value measured by one-way Anova with Tukey’s multiple comparison test; ^*^P < 0.05; ^**^P < 0.01; ^***^P < 0.001; ^****^P < 0.0001; ns, non-significant.

Using Seahorse technology, we found that the extracellular acidification rate (ECAR) was higher when Lin^−^ cells were exposed to ALL EVs (*P* < 0.0001, Fig. 8A). The same technology was used to quantify the oxygen consumption rate (OCR), revealing an increase in basal respiration (*P* < 0.001 for T-ALL and *P* < 0.01 for B-ALL) and in maximal respiration (*P* < 0.0001 for T-ALL and *P* < 0.001 for B-ALL) following the exposure of Lin^−^ cells to ALL EVs for 24 h (Fig. 8B). Furthermore, mitochondrial oxydoreductase activity improved (*P* < 0.001 for T-ALL and *P* < 0.0001 for B-ALL), while there was no change in the levels of reactive oxygen species (ROS) (Fig. S13D). Using flow cytometry and MitoTracker, we quantified the mitochondria levels expressed by late progenitors (LK cells), early progenitors (LSK cells) and HSC (LSK CD34^−^ cells). The relative quantity of mitochondria detected in these cells’ populations was unchanged after 24 h of exposure to ALL EVs (Fig. S13E). There was also no change in the expression levels of six proteins involved in mitochondrial energy metabolism (Fig. S13F). The mitochondrial potential measured with TMRM was lower for primitive HSPC than for late progenitors or total Lin^−^ cells (Fig. S13G). Interestingly, the mitochondrial membrane potential measured by flow cytometry following TMRM staining increased significantly for HSPC (LSK cells; *P* < 0.001) and HSC (LSK CD34^−^ cells; *P* < 0.001), when Lin^−^ cells were treated with ALL EVs (Fig. 8C). In conclusion, within 24 h, ALL EVs had enhanced the mitochondrial energy metabolism among primitive HSPC. We consequently established that ALL EVs were mainly responsible for mitochondrial activation and the loss of HSPC quiescence, which conduct to exhaustion of this population of cells.

## Discussion

The distinct metabolic profile of HSC reflected their location in a hypoxic niche [58, 59]. Fatty acid oxidation has been associated with HSC, determining whether they undergo symmetric or asymmetric cell division [60]. Studies on wild type rodent models have shown that diet regimen and high-fat diet-induced obesity triggers significant perturbations in HSC and homeostasis of the hematopoietic system [45, 61–65]. Other mice models have characterized the induced proliferation and mobilization of mouse HSPC following hypercholesterolemia [56, 57, 66, 67] or alterations in the cholesterol efflux pathways [68, 69]. Our study provided evidence that ALL EVs enhanced the mitochondrial activity in targeted HSPC, which affected their quiescence/maintenance. Mitochondrial metabolism has been described as an important regulator of HSC quiescence and self-renewal [70, 71]. Inhibition of the mitochondrial metabolism helped to maintain mouse HSC in culture ex vivo, and decreased their differentiation [72]. Metabolic regulation by the mitochondrial phosphatase was required for HSC differentiation [73]. Mitochondrial potentiation ameliorated age-related heterogeneity in HSC, which proved the importance of the mitochondrial mediation in the maintenance of HSC function [74]. There was also evidence that LDL was able to transfer cholesterol to HSC, which affected their quiescence/maintenance [56, 57] and accumulation of cholesterol occurred in EVs [75]. TGFβ [45, 50–52, 76] and ROS [77–79] have also been described as determinants for the regulation of HSC quiescence. However, we found here that ALL EVs have not affected neither TGFβ signaling nor ROS production in the primitive hematopoietic cells.

Mice models were useful to study how EVs produced by leukemia cells can disrupt the behavior of normal HSPC [12, 14, 18–20, 80]. In our study, we provided evidence that, in PDX models, EVs produced by ALL cells can disturb the homeostasis of HSPC in vivo. While the role of EVs is likely to remove excess and unnecessary constituents from cells to maintain cellular homeostasis [81], here we propose a new mechanism, suggesting that ALL EVs, probably because they were produced by highly proliferative leukemia cells, contained high levels of several metabolites. We discovered that cholesterol was the most relevant metabolite found highly represented in ALL EVs. Indeed, the lipid fraction isolated by Folch’s method from ALL EVs accelerated the mitochondrial metabolism and the loss of quiescence/maintenance in targeted healthy HSPC. While in our study, around 40% of ALL EVs expressed HSP70, the increase presence of detected metabolites might only represent a portion of ALL EVs. Indeed, we believe that the HSP70 negative EVs might be composed mostly by murine EVs, which may still be produced by the murine BM microenvironment during ALL development. This hypothesis was corroborated by our proteomic data analysis, while we have detected murine proteins among ALL EVs.

In conclusion, the transplantation of human ALL cells into immunodeficient NSG mice to generate PDX models to study T-ALL and B-ALL in vivo led to the discovery that ALL cells produced EVs that target endogenous murine HSPC in BM, disturbing their quiescence and maintenance. Our metabolomics study revealed that ALL EVs were enriched with several metabolites. They were particularly enriched with cholesterol, which improved the mitochondrial potential in targeted HSPC. All in all, this study highlights a new oncogenic EVs-dependent mechanism by which proliferative cancer ALL cells induced the exhaustion of quiescent healthy HSPC to compromise the hematopoietic system balance.

## Materials and methods

### Establishment of xenograft models

The ethics committee for animal welfare of the University of Burgundy and the French ministry of higher education and research approved all animal experiments (under reference APAFIS#16187-2018071914379464v3). We confirm that all experiments were performed according to the relevant guidelines and regulations of this committee. NOD/SCID/γc^−/−^ (NSG) mice (Charles River Laboratories, Lyon, France) were bred and housed in pathogen-free conditions. PDX cells have been established in a previous study [37]. Regarding cytogenetic characterization, T-ALL cells transplanted into PDX mice contain a STIL (SCL/TAL1 interrupting locus), as well as deletions in LEF1 and CDKN2A genes. Transplanted B-ALL cells, displayed a translocation in t(2;8) (p11;q24) MYC/IGK, as well as deletions in CDKN2A and RB1 genes. To induce leukemia in mice, we injected 5 × 10^5^ T-ALL or 10^5^ B-ALL cells in a volume of 300 µl of PBS1×, into the tail vein of non-irradiated 7–16-week-old male and female NSG mice. Males and females were randomly allocated to experimental groups and no blinding method was used for injection. There was no animal exclusion criteria. Mice were euthanized when they developed ALL symptoms. Mice were sacrificed at day 35 following the transplantation.

### Purification of EVs

When mice developed ALL symptoms, bones (tibias and femurs) from the two bottom legs were crushed in a mortar in 2 mL of PBS1×. The same procedure was followed for control NSG mice. Cells were removed after centrifugation (500 *g*, 5 min) and the supernatant was recovered for another centrifugation (10,000 *g*, 5 min) to remove cell debris. The supernatant was recovered, and then we pulled-down EVs by using the total EV isolation kit (15254394, Invitrogen) used at 10% (v/v), mixed by inverting the tubes several times and incubated for 15 min at 4 °C followed by a centrifugation (10,000 *g*, 10 min). The pellet of EVs was reconstituted in 200 µl of filtrated PBS1×. EVs were evaluated for their size and concentration by nanoparticle tracking analysis (NTA) using a NanoSight NS300 Instrument (Malvern Instruments, Malvern, England) and conserved at −80 °C. EVs were furthermore observed by TEM. The HSP70 peptide aptamer [26] was conjugated with the ATTO488 dye (ATTO488 NHS ester, Merck, Darmstadt, Germany) overnight, then Zebra Spin Desalting Column, Thermo Fisher Scientific, Illkirch-Graffenstaden, France) was used to remove excess dye. To make fluorescent EVs, HSP70-ATTO488 was administrated for 30 min before the EV pull-down step. EVs were characterized by flow cytometry on low SSC and FSC gated particles. EVs were then observed by fluorescence microscopy.

### EVs tested on primitive hematopoietic cells ex vivo

Bones (tibias and femurs) from the two bottom legs were crushed in a mortar in 10 mL of PBS1×. Total BM cells were then filtered with a sterile cell strainer (70 µm) and Sca1^+^ cells (130-123-124, Miltenyi Biotec, Paris, France) or Lin^−^ cells (130-110-470, Miltenyi Biotec) were isolated. Cells were cultured in 96-well U-bottom plates with 200 µL of the StemMACS media (Miltenyi Biotec), supplemented with PSA (Pan Biotech, Aidenbach, Germany), murine Stem cell factor (SCF, 25 ng/mL, 130-101-741, Miltenyi Biotec), murine Interleukin 3 (IL3, 10 ng/mL, 130-096-687, Miltenyi Biotec), and murine Interleukin 6 (IL6, 10 ng/mL, 130-096-682, Miltenyi Biotec). Cells were divided into four wells (Mock, NSG EVs, T-ALL EVs, B-ALL EVs) and treated with EVs, 10^9^ particles (Sca1^+^ cells) or 2 × 10^9^ particles (Lin^−^ cells). After exposure with EVs during 24 h, we tested the differentiation capacity of Sca1^+^ cells using CFU assays. Using flow cytometry (gating on LSK), we tested the effect of 24 h exposure of Lin^−^ cells to EVs on apoptosis by Annexin V staining, on the cell cycle activity following Ki67 staining, on the expression of primitive markers (SLAM, CD34^−^ cells), on the TGFβ pathway by phospho-Smad2/3 staining, and finally on the mitochondrial activity following labeling with MitoTracker Green FM (Thermo Fisher Scientific) or Tetramethylrhodamine methyl ester (TMRM, Thermo Fisher Scientific). To examine EVs cells’ intake, we treated total BM cells, Lin^−^ or Sca1^+^ cells with HSP70-ATTO488 EVs for 2 h in PBS1× at 4 °C. We used 10^9^ particles (Sca1^+^ cells) or 2 × 10^9^ particles (Lin^−^ cells and total BM cells). Following washing with PBS1×, cells were stained with specific antibodies to analyze primitive hematopoietic populations. The uptake of EVs cells was analyzed by flow cytometry for ATTO488 florescence on specific subpopulations (LSK, SLAM). Sca1^+^ cells were treated for 2 h with methyl-β-cyclodextrine (Merck) at 2.5 mM, prior to staining cells with CTB, and the uptake capacity of EVs was assessed following the same procedure.

### Injection of EVs in NSG mice

EVs isolated from NSG controls or ALL PDX mice were i.v. injected into the tail vein, three times every 10 days at a dose of 10^10^ particles in 300 µL PBS1×. Ten days after the third injection, we recovered the BM cells. Bones (tibias and femurs) from the two bottom legs were crushed in a mortar in 10 mL of PBS1×. Total BM cells were then filtered with a sterile cell strainer (70 µm), and Sca1^+^ cells (130-123-124, Miltenyi Biotec) were isolated and counted. For the transplantation study, 3 × 10^5^ Sca1^+^ cells were i.v. injected in C57BL/6.SJL (Ly.1) mice (Charles River Laboratories). Recipient mice were pretreated, one and two days before the transplantation, with intraperitoneal injections of 20 mg/kg busulfan (Merck). Four weeks after the transplantation, we assessed hematopoietic reconstitution with specific antibodies by flow cytometry, in BM. EVs isolated from NSG controls or ALL PDX mice were also i.v. injected into the tail vein of NSG mice, three times every 10 days at a dose of 10^10^ particles in 300 µL PBS1×. Ten days after the third treatment, we injected 5 × 10^5^ T-ALL cells or 10^5^ B-ALL cells and studied the development of ALL by measuring the percentage of T-ALL or B-ALL cells in different organs by flow cytometry at day 35.

### Metabolism

Lin^−^ cells divided into four wells (Mock, NSG EVs, T-ALL EVs, B-ALL EVs) were treated for 24 h with EVs (2 × 10^9^ particles) in media with murine cytokines. Following treatment, Lin^−^ cells were seeded on specific 96-well plates (Seahorse Bioscience, Agilent Technologies, Les Ulis, France) pre-coated with Cell-Tak (Thermo Fisher Scientific) for the binding of Lin^−^ cells. The media was changed for Seahorse XF RPMI media, pH 7.4 (Seahorse Bioscience, Agilent Technologies). Oxygen consumption and extracellular acidification were measured using a metabolic flux analyzer (Seahorse Bioscience, Agilent Technologies) under basal conditions and in the presence of the mitochondrial inhibitor oligomycin (1.5 μM), the uncoupling compound carbonylcyanide-4-(trifluoromethoxy)phenylhydrazone (FCCP, 1 μM), the respiratory chain inhibitors rotenone (0.5 μM), and antimycin A (0.5 μM) from the Mito Stress Test (Agilent Technologies). XTT assay (CyQUANT XTT Cell Viability Assay, Thermo Fisher Scientific) was used to quantify the mitochondrial redox status of Lin^−^ cells following 120 min of exposure with reagents; absorbances were measured at 450 nm and 660 nm. Amplex Red Hydrogen Peroxide/Peroxidase Assay Kit (Thermo Fisher Scientific) was used to measure the peroxide hydrogen (ROS) in Lin^−^ cells, absorbance was measured at 560 nm, following 90 min with reagents. Tetramethylrhodamine methyl ester (TMRM) (Thermo Fisher Scientific) was used to measure changes in the mitochondrial membrane potential by flow cytometry. Lin^−^ cells were treated with Sodium pyruvate solution (S8636, Merck), LDL (LP2-2MG, 6.7 mg/mL, Merck) containing cholesterol (14.03 mg/mL), D-( + )-glucose (Merck), ATP (A1852, Merck) and amino acid standard solution (AA-S-18, Merck), at the doses indicated in the Figure legends.

### Hematopoietic and mesenchymal colony-forming unit (CFU) assays

After exposure of Sca1^+^ cells treated with EVs for 24 h, 200 µl of the culture was added to semisolid methylcellulose media supplemented with growth factors (Methocult M3434; Stem Cell Technologies, Saint-Egrève, France) at 37 °C in 98% humidity and 5% CO_2_ for 10 days. Hematopoietic CFU were counted at the days indicated in figure legends. NSG mice developing ALL (injected with ALL cells) or injected with EVs were processed with similar procedures. Bones (tibias and femurs) from the two bottom legs were crushed in a mortar in 10 mL of PBS1× and supplemented with 100 µL of collagenase A (10% w/v) (Merck), and cells were incubated at 37 °C in 98% humidity and 5% CO_2_ for 2 h. Total BM cells were then filtered with a sterile cell strainer (70 µm) and Sca1^+^ cells were isolated with magnetic beads (130-123-124, Miltenyi Biotec). Sca1^+^ cells were counted with Trypan blue (Thermo Fisher Scientific). Twenty-five percent of the Sca1^+^ were added to semisolid methylcellulose media supplemented with growth factors (Methocult M3434; Stem Cell Technologies) at 37 °C in 98% humidity and 5% CO_2_ for 7 days. The remaining 75% of the Sca1^+^ cells were cultured in the mouse MesenCult Expansion kit (Stem Cell Technologies) for growth of mesenchymal cells for up to 2 weeks. Cells were stained with crystal violet to count the mesenchymal CFU at day 14. For the in vivo experiment, after the three i.v. injections of EVs in NSG mice, Sca1^+^ cells were recovered from the BM of mice and counted. Sca1^+^ cells (10^5^ Sca1^+^ cells) were added to M3434 semisolid methylcellulose media, for 7 days. Total CFU and CFU-GM per Sca1^+^ cells plated in the semi solid media was then calculated. Distribution among CFU-GM, CFU-G, CFU-M and BFU-E was also measured.

### Flow cytometry and fluorescent-activated cell sorting (FACS)

Bones, tibias and femurs from the two bottom legs were crushed in a mortar in PBS1× and total BM cells were filtered with a sterile cell strainer (70 µm). Spleens were also crushed and filtered with the sterile cell strainer in a hemolysis solution, and the cells were washed with PBS1×. The development of T-ALL and B-ALL in NSG mice was characterized in BM by flow cytometry using the following anti-human antibodies: anti-hCD45-APC (1:100, 130-110-633, Miltenyi Biotec), anti-hCD7-VioBright-FITC (1:100, 130-123-864, Miltenyi Biotec) for T-ALL and anti-hCD19-VioBright-B515 (1:100, 130-113-650, Miltenyi Biotec) for B-ALL detection. These antibodies were also used to distinguish between human ALL cells and murine cells. Lin^−^ mouse BM cells (130-110-470, Miltenyi Biotec), c-Kit^+^ mouse BM cells (130-091-224, Miltenyi Biotec), Sca1^+^ mouse BM cells (130-123-124, Miltenyi Biotec) or hCD45^+^ ALL cells (130-045-801, Miltenyi Biotec) were magnetically purified using LS columns (Miltenyi Biotec). We used the following antibodies; Sca1-APC-Cy7 (1:100, 108126, Biolegend, Paris, France), Sca1-VB-FITC (1:100, 130-116-490, Miltenyi Biotec), CD48-PE-Cy7 (1:100, 560731, BD Biosciences, Le Pont de Claix, France), CD48-FITC (1:100, 103404, Biolegend), CD48-APC (1:100, 130-124-727, Miltenyi Biotec), c-Kit-BV650 (1:100, 135125, Biolegend), c-Kit-PE-Cy7 (1:100, 558163, BD Biosciences), CD150-PerCP-Cy5.5 (1:100, 115921, Biolegend), murine CD45.2-Vioblue (1:100, 130-102-980, Miltenyi Biotec). AF555-conjugated cholera toxin subunit B (CTB, 1 µg/mL, C-34776, Thermo Fisher Scientific) were used to stain lipid rafts. The peptide aptamer which allows to detect HSP70 [26], was conjugated with the ATTO488 dye (ATTO488 NHS ester, Merck). HSP70-ATTO488 (1 mg/mL) was used on permeabilized or unpermeabilized cells (dilution 1:100) to reciprocally detect intracellular HSP70 and membrane-anchored HSP70 on the cell surface. We used MitoTracker Green FM dye (Thermo Fisher Scientific) and Tetramethylrhodamine methyl ester (TMRM, Thermo Fisher Scientific) 30 min before flow cytometry recording of Lin^−^ cells. After cell surface staining, cells were fixed and permeabilized using BD Cytofix/Cytoperm Plus Fixation/ Permeabilization Kit (BD Biosciences). Viability was assessed with Hoechst 33342 (1:1,000, Thermo Fisher Scientific), 7-AAD (1:50, 559925, BD Biosciences) or with a Fixable Viability Stain (FVS) 450, 520 or 575 (1:1,000, BD Biosciences). For studies on cell cycle and apoptosis, we used anti-Ki67-BV421 antibody (1:50, 562899, BD Biosciences), anti-Ki67-FITC antibody (1:20, 556026, BD Biosciences), 7-AAD (1:50, 559925, BD Biosciences) and anti-Annexin-V-FITC (1:50, 640906, Biolegend). Anti-p-Smad2 (S465/S467)/p-Smad3 (S423/S425)-PE-CF594 antibody (1:100, 562697, BD Biosciences) was used after cell surface staining and fixation/permeabilization. We also used anti-p-Stat3 (Y705)-PE-CF594 (1:100, 562673, BD Bioscience), anti-p-Akt (S473)-BV421 (1:100, 562599, BD Bioscience) and anti-p-Stat5 (Y694)-PE-Cy7 (1:100, 560117, BD Bioscience). To separate NSG donor cells (Ly.2) from recipient cells in C57BL/6.SJL (Ly.1) mice, we used the anti-CD45.2-Vioblue (1:100, 130-102-980, Miltenyi Biotec) antibody. The in vivo human hematopoietic reconstitution was analyzed with the following antibodies; anti-hCD45-APC (1:100, 130-110-633, Miltenyi Biotec), anti-hCD33-PE-Cy5 (1:10, 551377, BD Pharmingen) and anti-hCD19-VioBright-B515 (1:100, 130-113-650, Miltenyi Biotec). Cell subsets were analyzed using a LSR-Fortessa (BD Biosciences). Cells were sorted on a FACS Aria cell sorter (BD Biosciences) equipped with BD FACSDiva software (BD Biosciences). Data were analyzed using FlowJo software (V10, TreeStar Inc, Ashland, USA).

### Fluorescence microscopy

PDX mice transplanted with T-ALL or B-ALL cells were euthanized when they developed symptoms of ALL. Hind limb bones were collected, stripped of soft tissue, fixed and decalcified in a specific buffer (#3800400, Decalcifier-I, Leica, Paris, France) for 48 h, processed and embedded in paraffin. Thick sections were cut from paraffin-embedded samples and used for fluorescent staining with the HSP70-ATTO488, rabbit anti-hCD7 (1:200, ab109296, Abcam, Paris, France) or rabbit anti-hCD19 (1:200, SAB5500047, Merck), followed by anti-rabbit AF568 antibody (Thermo Fisher Scientific). The cells extracted from BM were stained with anti-hCD45-APC (1:100, 130-110-633, Miltenyi Biotec) and permeabilized using BD Cytofix/Cytoperm Plus Fixation/Permeabilization Kit (BD Biosciences) and then stained with Ki67-BV421 antibody (1:50, 562899, BD Biosciences) and 7-AAD (1:50, 559925, BD Biosciences) and the HSP70-ATTO488 dye prior to FACS. The cells extracted from BM were stained with anti-hCD45-APC (1:100, 130-110-633, Miltenyi Biotec) and HSP70-ATTO488, FACS was used for cell sorting, and cells were deposited on a glass slide (Superfrost plus, Thermo Fisher Scientific) for up to 10 min. After staining with HSP70-ATTO488, EVs were pulled-down and pellets were reconstituted in PBS1×, and then EVs were deposited on a glass slide for up to 10 min. After the staining of lipid rafts on Lin^−^ cells with AF555-conjugated cholera toxin subunit B (CTB, 1 µg/mL, C-34776, Thermo Fisher Scientific), Lin^−^ cells were incubated for 2 h with HSP70-ATTO488-stained EVs, then washed and applied on a glass slide for up to 10 min. Cells and bone sections were fixed with ProLong Gold Antifade reagent containing DAPI (P36931, Thermo Fisher Scientific). Fluorescent images were acquired with an Axio Imager M2 (Zeiss, Marly-le-Roi, France). Fluorescent optical sections of cells were obtained under magnification ×63, using an Axio Imager M2 (Zeiss) coupled with an Apotome.2 (Zeiss). Fluorescent images were processed for study (Fiji, NIH software, USA).

### Analysis of EV cargoes

We used Folch’s method to purify the lipid sub-fraction. Briefly, EVs were reconstituted in NaCl_2_, CHCl_3_ and methanol. After agitation for 2 h, NaCl_2_ was added, agitated for 2 min, left at room temperature for 5 min and centrifuged for 5 min. The upper phase was aspirated and the lower phase transferred to a Pyrex vial for drying. The lipid sub-fraction was reconstituted in DMSO and micelles, obtained by diluting lipid fraction tenfold in PBS1× at room temperature for 20 min, were applied to Lin^−^ cells for 24 h. For quantification of cholesterol, we used the Indiko Clinical Chemistry Analyzer (Thermo Fisher Scientific). For the glucose oxidase assay, we used the Glucose GOD FS kit (DiaSys Diagnostic Systems GmbH, Holzheim, Germany) and D-( + )-Glucose (Merck) as the standard for quantification. ATP was measured with the Cell Titer Glo (Promega, Charbonnières-les-Bains, France) and standard (A1852, Merck) for the quantification.

### Statistics

All data were expressed as means ± standard deviation (SD). Differences between two groups were assessed with the two-tailed Student’s unpaired *t* test. The one-way Anova with Tukey’s multiple comparison test was used to assess differences between more than two groups. No statistical methods were used to predetermine the sample size. Mice were randomly allocated to experimental groups. No blinding method was used for injection. There was no animal exclusion criteria. The variance was similar between the groups that were being statistically compared. Statistics were performed using Prism 6 (GraphPad, San Diego, USA) and significance is indicated in the figures.

For more information about global lipidomic profiling on EVs, UPLC proteomic analysis on EVs by LC-MS/MS, UPLC amino acid analysis on EVs by LC-MS/MS, CD34^+^ cord blood isolation, treatment with EVs and transplantation in NSG mice, culture of ALL cells and hematopoietic murine cells on MS5 feeder cells, MS5 cells migration assay, Western blots, transmission electron microscopy and artificial proteoliposomes with HSP70 expression, see the supplementary Materials and Methods online.

## Supplementary information

Supplementary Figures and Table

Supplementary Materials and Methods

Reproducibility checklist

## Data Availability

All data generated or analyzed during this study are available from the corresponding author on reasonable request.
