# Supplementary material for: Acute lymphoblastic leukemia-derived extracellular vesicles affect quiescence of hematopoietic stem and progenitor cells
Source: Cell Death Dis. 2022 Apr 12;13(4):337. doi: 10.1038/s41419-022-04761-5 (PMC9005650; doi:10.1038/s41419-022-04761-5)
Supplement: Supplementary file 1 — Supplementary Figures and Table [file 41419_2022_4761_MOESM1_ESM.docx]

**Acute lymphoblastic leukemia-derived extracellular vesicles affect quiescence of hematopoietic stem and progenitor cells**

Aleksandra Georgievski,^1,2^ Anaïs Michel,^1^ Charles Thomas,^1,2^ Zandile Mlamla,^1,3^ Jean-Paul Pais de Barros,^1,2,3^ Stéphanie Lemaire-Ewing,^1,4^ Carmen Garrido^1,2,5^ and Ronan Quéré.^1,2,*^

^1^UMR1231, Inserm/Université Bourgogne Franche-Comté, Dijon, France.

^2^LipSTIC Labex, Dijon, France.

^3^Plateforme de Lipidomique Analytique, Université Bourgogne Franche-Comté, Dijon, France.

^4^Département de Biochimie, Hôpital Universitaire François Mitterrand, Dijon, France.

^5^Centre Georges François Leclerc-Unicancer, Dijon, France.

**Supplementary Figures S1 to S14**

**Supplementary Table S1**

**
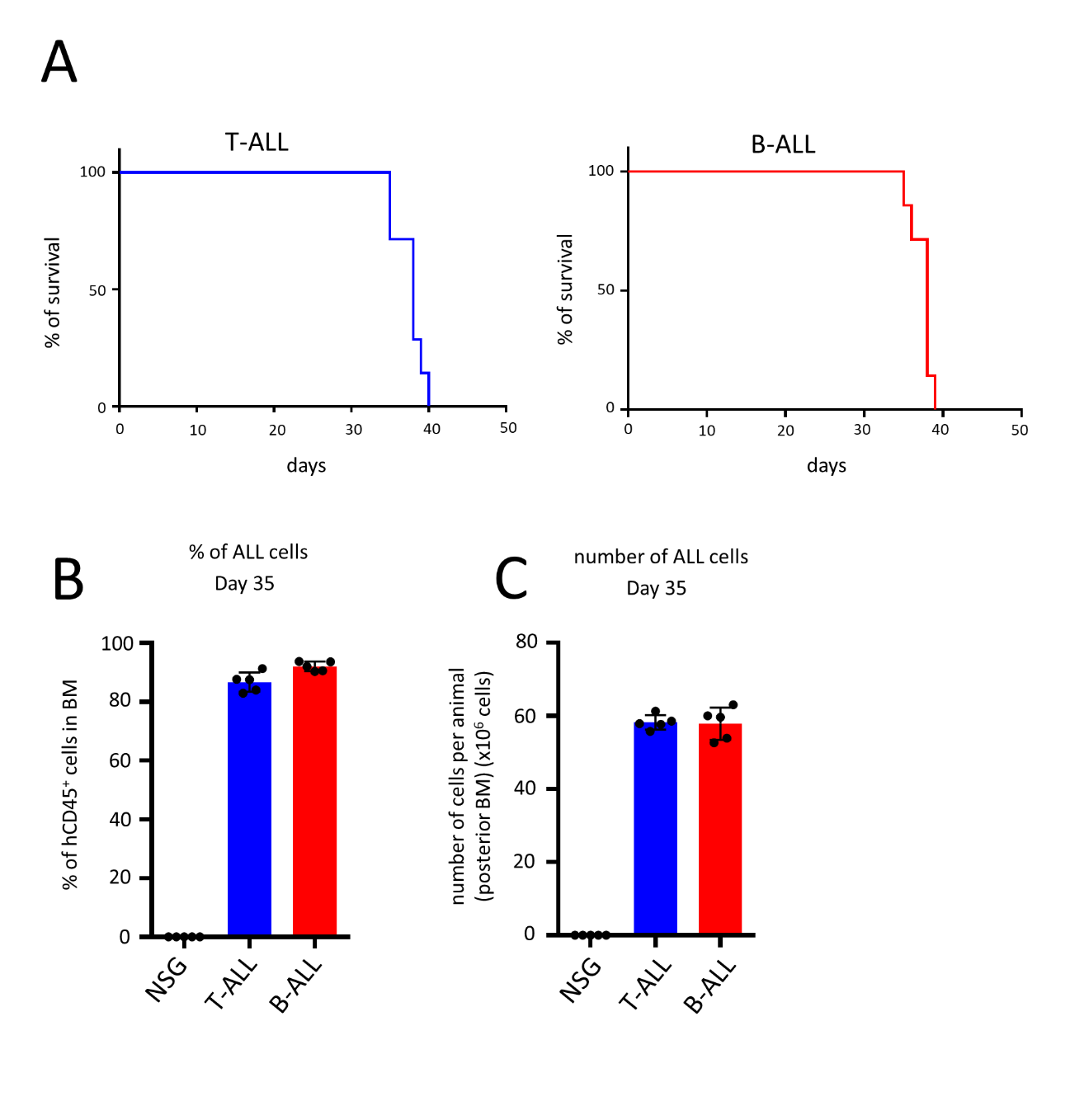
**

**Supplementary Fig. S1: Data showing the engraftment efficiency following the transplantation of 5×10^5^ T-ALL or 10^5^ B-ALL cells in NSG mice.**

**A** Survival curves following the transplantation (n=7 mice). **B** When mice were sacrificed at day 35 (n=5 mice), the BM contain >80% of leukemic cells as assessed by flow cytometry (hCD45^+^ cells). The absolute number of ALL cells detected in BM (n=5 mice) at day 35.

**
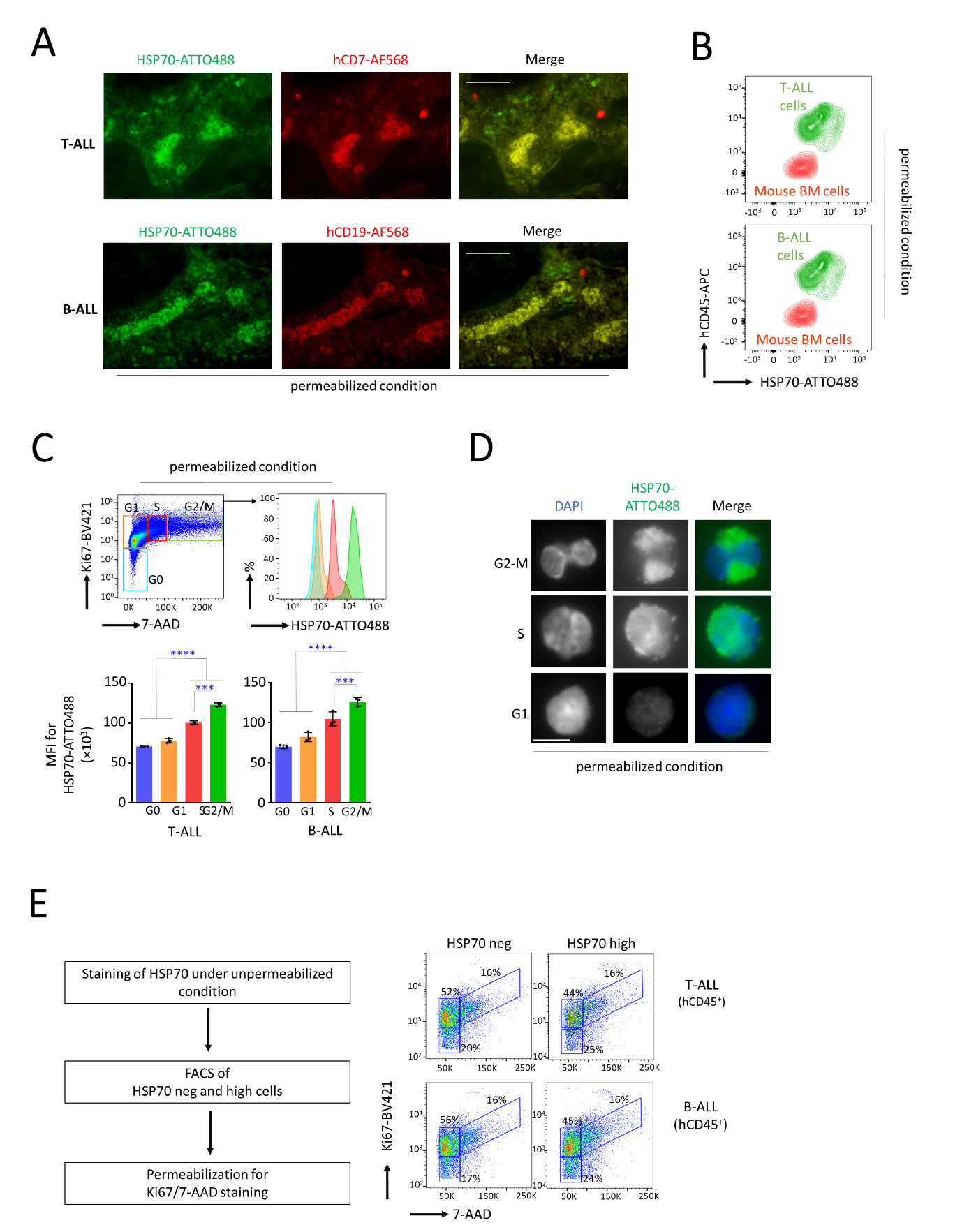
**

**Supplementary Fig. S2: PDX T-ALL and B-ALL cells expressed high levels of HSP70.**

**A** Immunostaining on bone sections showing that T-ALL (hCD7^+^) and B-ALL (hCD19^+^) cells expressed high levels of HSP70 in PDX mice. Experiment performed at day 35 following the i.v. transplantation of 5×10^5^ cells for T-ALL or 10^5^ cells for B-ALL. HSP70 is stained with HSP70-ATTO488, microscopy with magnification ×40, scale bars represent 100µm. **B** Flow cytometry showing that T-ALL and B-ALL cells (green) in the BM of PDX models expressed HSP70, while murine endogenous cells in BM (red) did not express HSP70. BM cells were permeabilized for HSP70 detection. BM at day 35 for T-ALL or B-ALL. **C** Flow cytometry showing that ALL PDX cells expressed marked levels of HSP70 during the active phase of the cell cycle. Staining with anti-Ki67 antibody and 7-AAD was performed on hCD45^+^ cells. Data are shown as means ± SD; n=3 mice. P value measured by one-way Anova with Tukey’s multiple comparison test; ***, P<0.001; ****, P<0.0001. **D** Immunostaining on fluorescent activated cells sorting (FACS) single cells showing a high detection of HSP70 in the cytoplasm of representative cells in S and G2/M phases of the cell cycle, microscopy with magnification ×63, scale bar represents 5µm. **E** HSP70 high and neg cells show similar cell cycle activity. ALL cells were stained for HSP70 expression under unpermeabilized condition. After FACS purification on hCD45^+^ cells of HSP70 neg and high, cells are permeabilized and stained with Ki67 and 7-AAD.


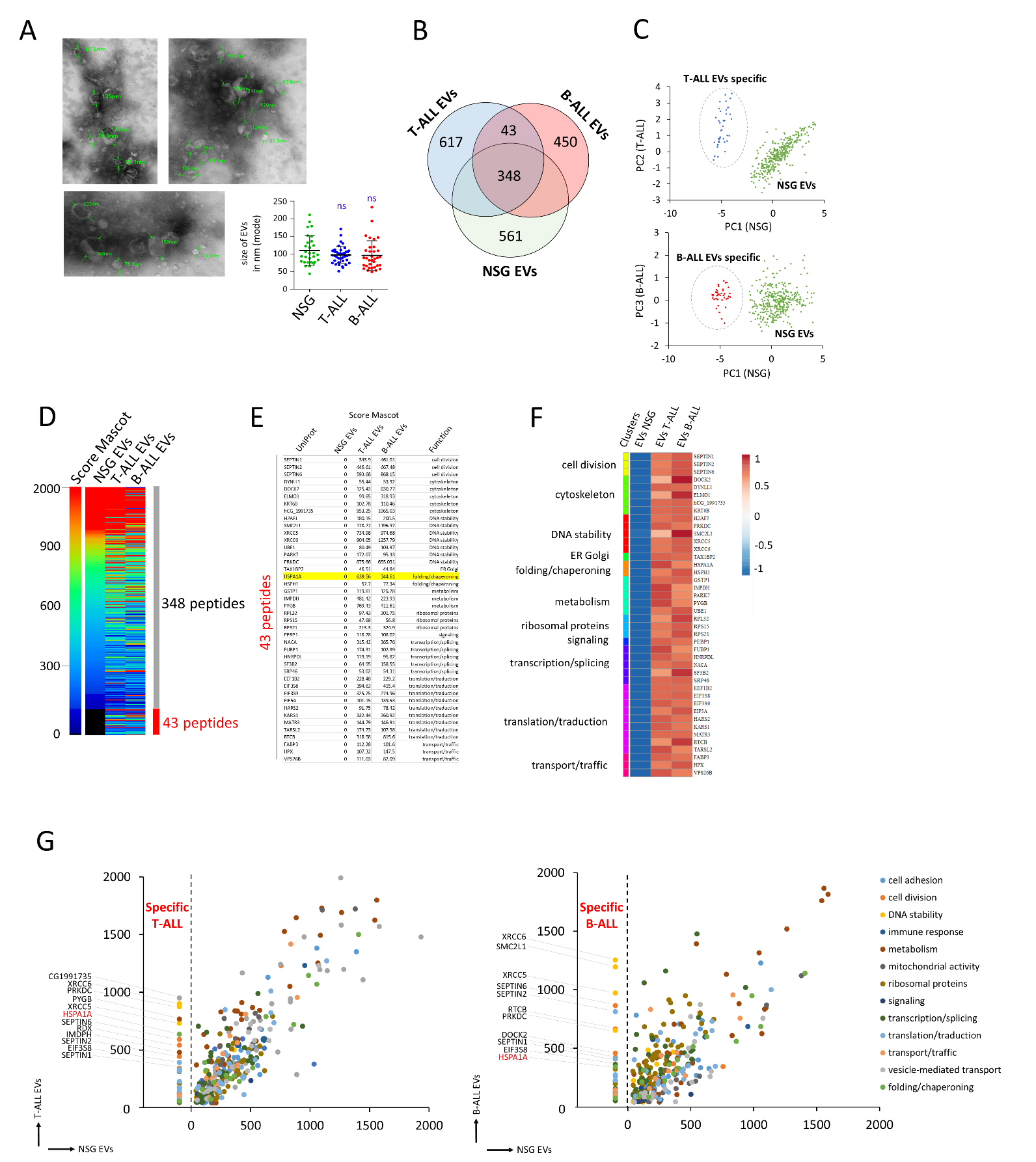


**Supplementary Fig. S3: Proteomic on ALL EVs.**

**A** TEM confirmed that size of EVs correspond more to large EVs (also called ectosomes). Examples of TEM images are shown on the left. Statistic on the bottom panel described mean ± SD; n>30 EVs measured. P value measured by one-way Anova with Tukey’s multiple comparison test; ns, non-significant. **B** Description of the proteins detected by mass spectrometry in order to find proteins specifically expressed by T-ALL and B-ALL EVs but not represented in NSG EVs (43 peptides), as well as peptides commonly found in all EVs (348 peptides). We focused our study on human proteins found commonly expressed in T-ALL and B-ALL EVs (391 proteins), and compared their level of expression to the endogenous murine proteins found in control NSG EVs. **C** Proteomic data examined with the Principal Component Analysis (PCA) plots (Score Mascot data were Ln transformed).  **D** Proteomic data analyzed with the Multi Experiment Viewer. Forty-three proteins were commonly detected only in ALL EVs and not found in NSG EVs. **E** List of proteins specifically detected in T-ALL and B-ALL EVs, but not found in NSG EVs. Quantification of the peptides (Score Mascot). In yellow is shown the inducible HSP70 protein (HSPA1A). **F** Heatmap of the 43 proteins and their biological functions (Score Mascot data were Ln transformed). **G** Scatter plot showing the quantity of proteins (Score Mascot) and their biological functions in T-ALL and B-ALL EVs, compared with proteins found in NSG EVs. On the left are all the proteins detected only in T-ALL and B-ALL EVs, such as HSP1A1 (red).

**
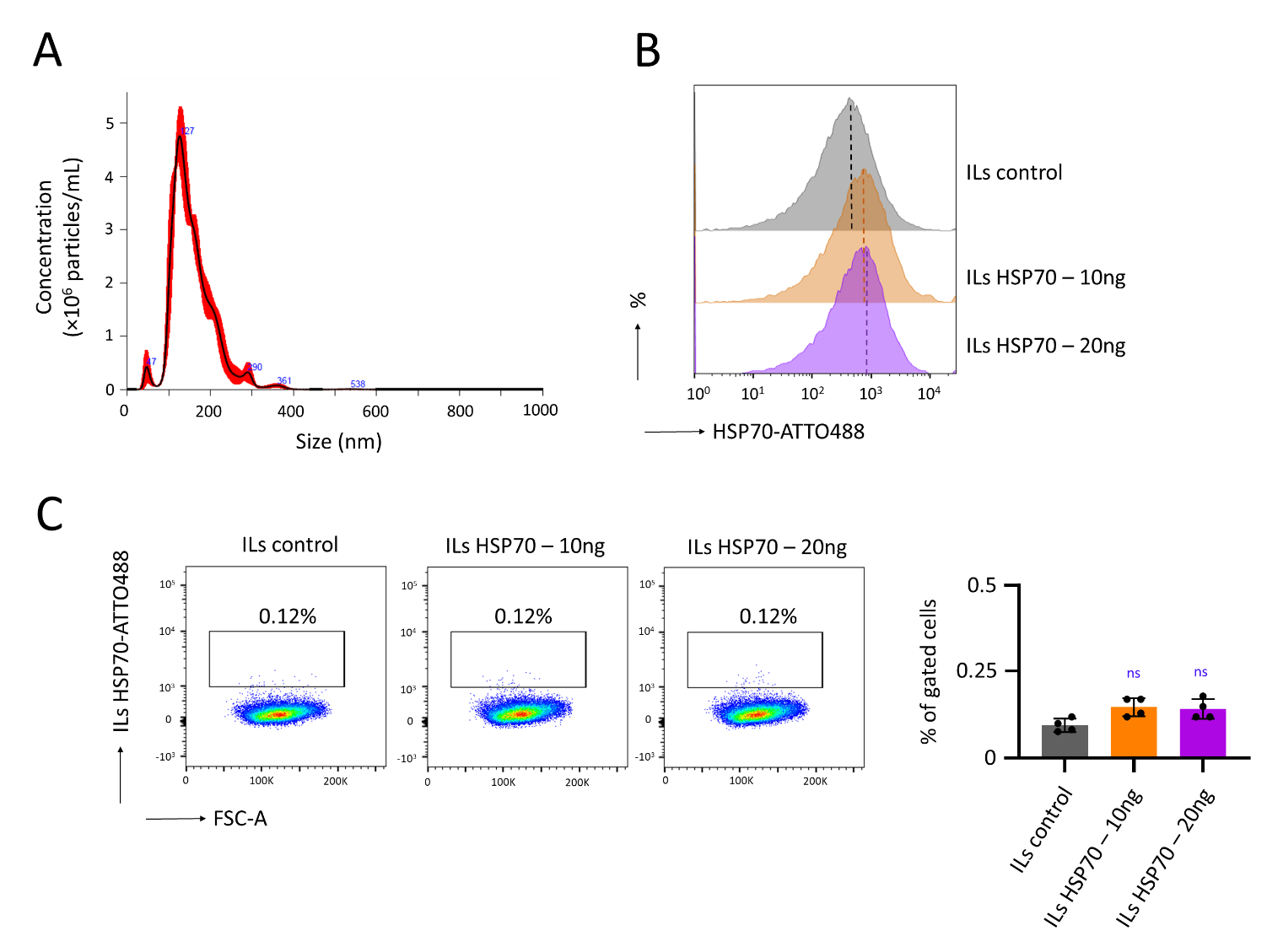
**

**Supplementary Fig. S4: Artificial lipid vesicles containing HSP70 failed to bind HSPC *in vitro*.**

**A** Nanoparticle tracking analysis (NTA) used to quantify the artificial lipid vesicles and to determine their size with a median pic calculated at 120nm. **B** Flow cytometry showing the expression of HSP70 on artificial lipid vesicles included with the recombinant HSP70 (HSP70-ATTO488). Artificial lipid vesicles without HSP70 inclusion were used as control. **C** Flow cytometry showing the non-binding of ATTO488-stained artificial lipid vesicles on Lin^-^ cells. The conformation of HSP70 can be changed when it is bound to artificial lipid vesicles, this conformational change might also consequently hinder the binding. Data are shown as mean ± SD; n=4 biological replicates. P value measured by one-way Anova with Tukey’s multiple comparison test; ns, non-significant

**
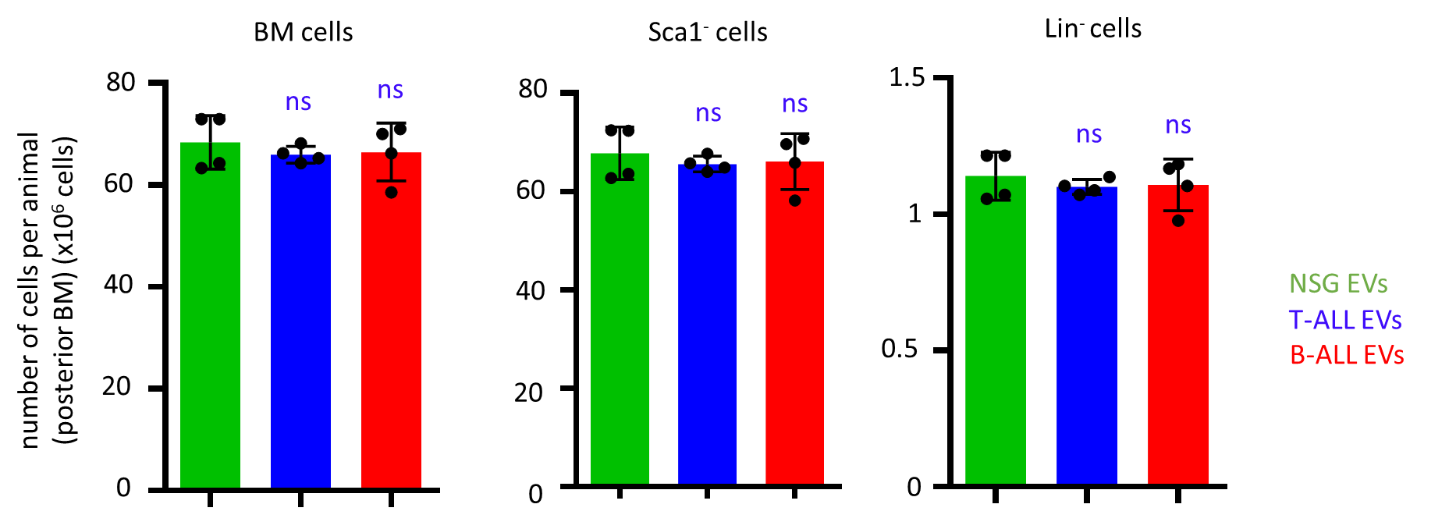
**

**Supplementary Fig. S5:** **Effect of the injection of ALL EVs *in vivo* on murine BM cells.**

Absolute number of total BM, Sca1^-^ and Lin^-^ cells detected in the BM of mice injected with NSG EVs, T-ALL EVs and B-ALL EVs. Data are shown as mean ± SD; n=4 mice. P value measured by one-way Anova with Tukey’s multiple comparison test; ns, non-significant

**
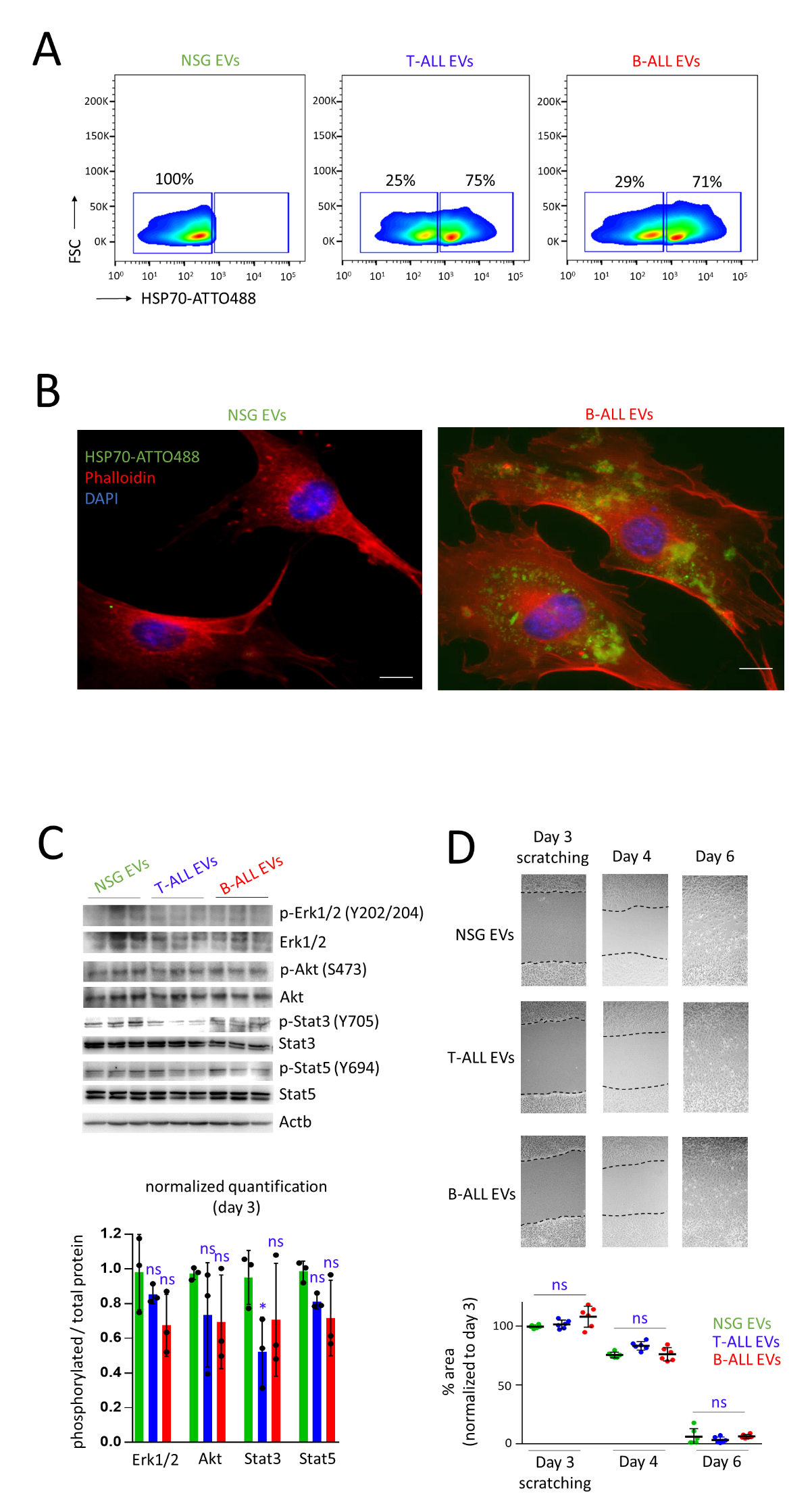
**

**Supplementary Fig. S6:** **ALL EVs penetrate in MS5 cells but do not modify their signaling and growing.**

**A** Flow cytometry on MS5 cells showing that >70% of the cells are positive following 24-hours exposure with fluorescent ALL EVs (ATTO488 EVs). **B** Examples of microscopy showing intake of fluorescent EVs (ATTO488 EVs) by murine MS5 cells. Magnification ×40, scale bar represents 25µm. **C** MS5 cells are treated with ALL EVs or control EVs (2×10^9^ particles) for 3 days and the phosphorylation of important proteins involved in further signaling are analyzed by western blot. Data are shown as mean ± SD; n=3 biological replicates. P value measured by one-way Anova with Tukey’s multiple comparison test; ns, non-significant. **D** Wound healing assay shows the non-acceleration in growth of MS5 cells, following their exposure to ALL EVs. Scratching are done 3 days after exposure of MS5 cells to ALL EVs or control EVs (2×10^9^ particles). Magnification ×4. Data are shown as mean ± SD; n=6 biological replicates. P value measured by one-way Anova with Tukey’s multiple comparison test; ns, non-significant.

**
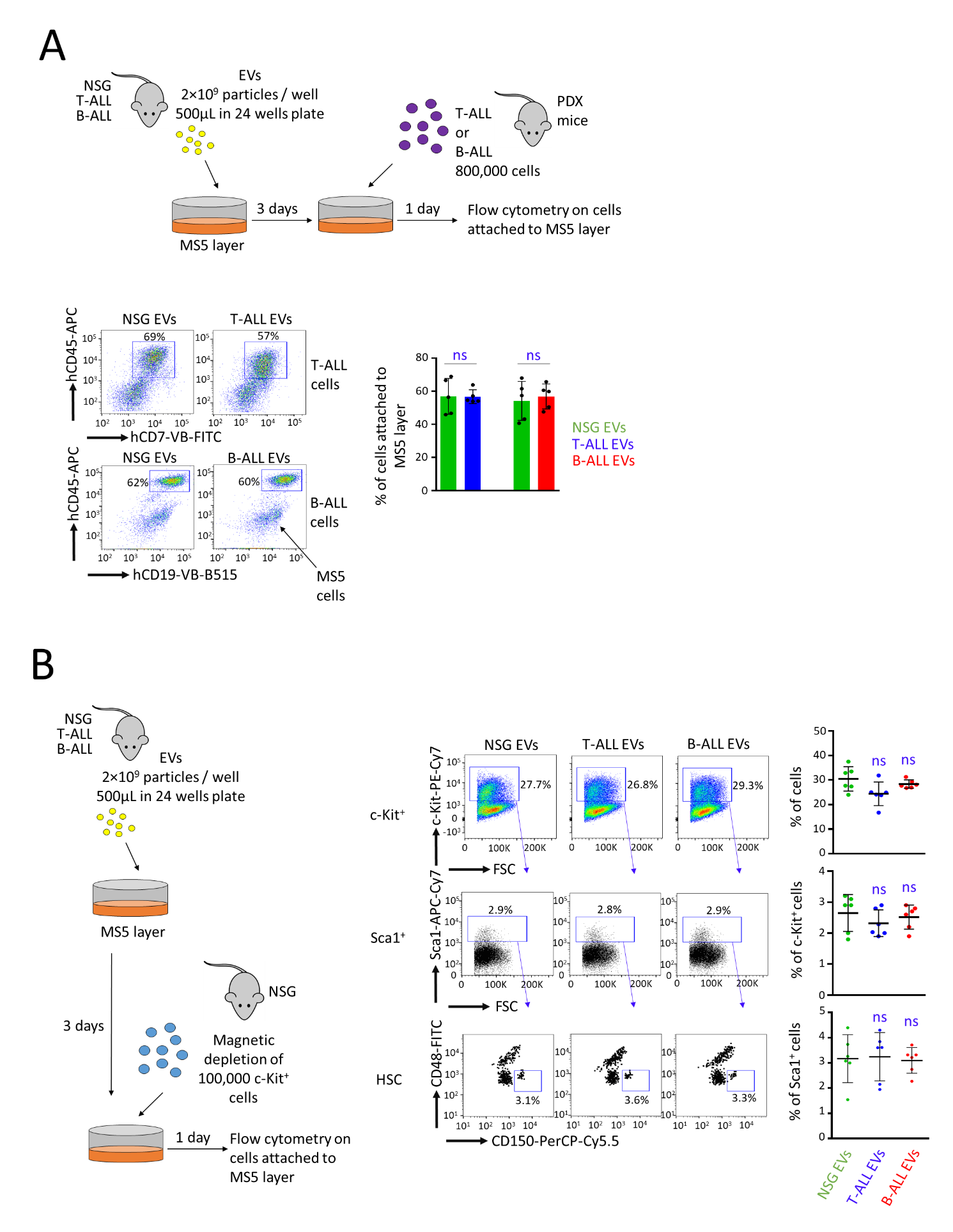
**

**Supplementary Fig. S7:** **ALL EVs do not modify the MS5 capacity to bind ALL cells or murine primitive hematopoietic cells *in vitro*.**

**A** Procedure followed to measure the MS5 capacity to bind ALL cells, after an exposure to ALL EVs (2×10^9^ particles) for 3 days. The percentage of T-ALL (hCD45^+^ hCD7^+^) cells or B-ALL (hCD45^+^ hCD19^+^) cells is assessed by flow cytometry one day after being applied on MS5 cells. Data are shown as mean ± SD; n=5 biological replicates. P value measured by two-tailed Student’s unpaired t-test; ns, non-significant. **B** Procedure followed to measure the MS5 capacity to bind murine c-Kit^+^ cells, after an exposure to ALL EVs (2×10^9^ particles) for 3 days. The percentage of c-Kit^+^ cells, Sca1^+^ cells and HSC (SLAM; CD150^+^ CD48^-^) is assessed by flow cytometry one day after c-Kit^+^ cells are applied on MS5 cells. Gating strategy used is shown. Data are shown as mean ± SD; n=6 biological replicates. P value measured by one-way Anova with Tukey’s multiple comparison test; ns, non-significant.

**
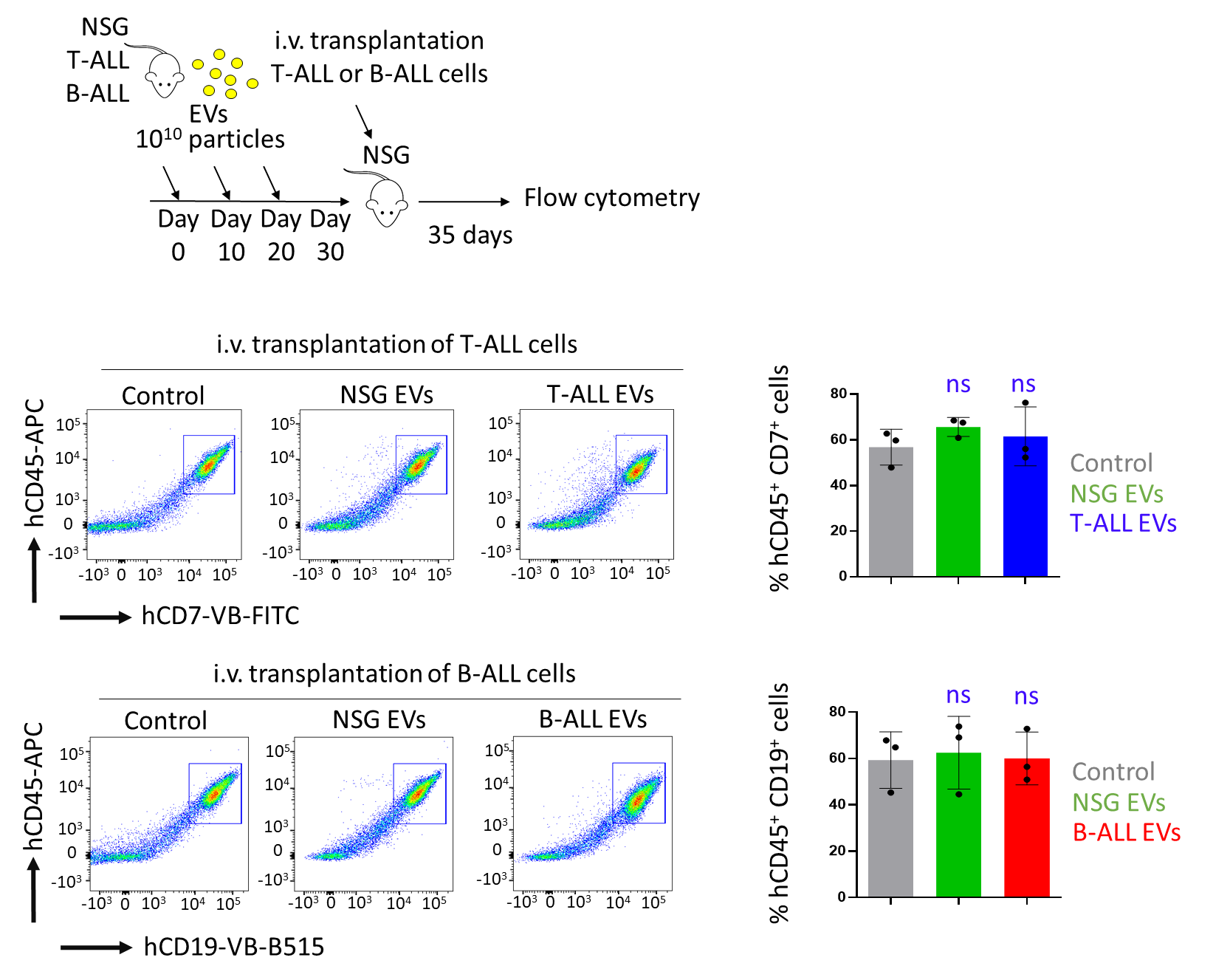
**

**Supplementary Fig. S8:** **ALL EVs injected *in vivo* do not affect ALL development in PDX mice.**

NSG mice are treated with ALL EVs or control EVs, through three consecutive i.v. injections, at 10^10^ particles / mouse / every 10 days. Ten days after the third injection, we injected mice with 5×10^5^ T-ALL cells or 10^5^ B-ALL cells. Mice are then sacrificed, when they start to develop ALL disease, 35 days after the ALL cells injections. Flow cytometry showing that T-ALL (hCD7^+^ hCD45^+^) and B-ALL (hCD19^+^ hCD45^+^) cells were similarly detected in BM for each groups of mice. This experiment show that pretreatment with ALL EVS is not accelerating or blocking the development of ALL, while the same quantity of ALL cells is observed in BM. T-ALL on the top panel and B-ALL on the bottom panel. Data are shown as mean ± SD; n=3 mice. P value measured by one-way Anova with Tukey’s multiple comparison test; ns, non-significant.

**
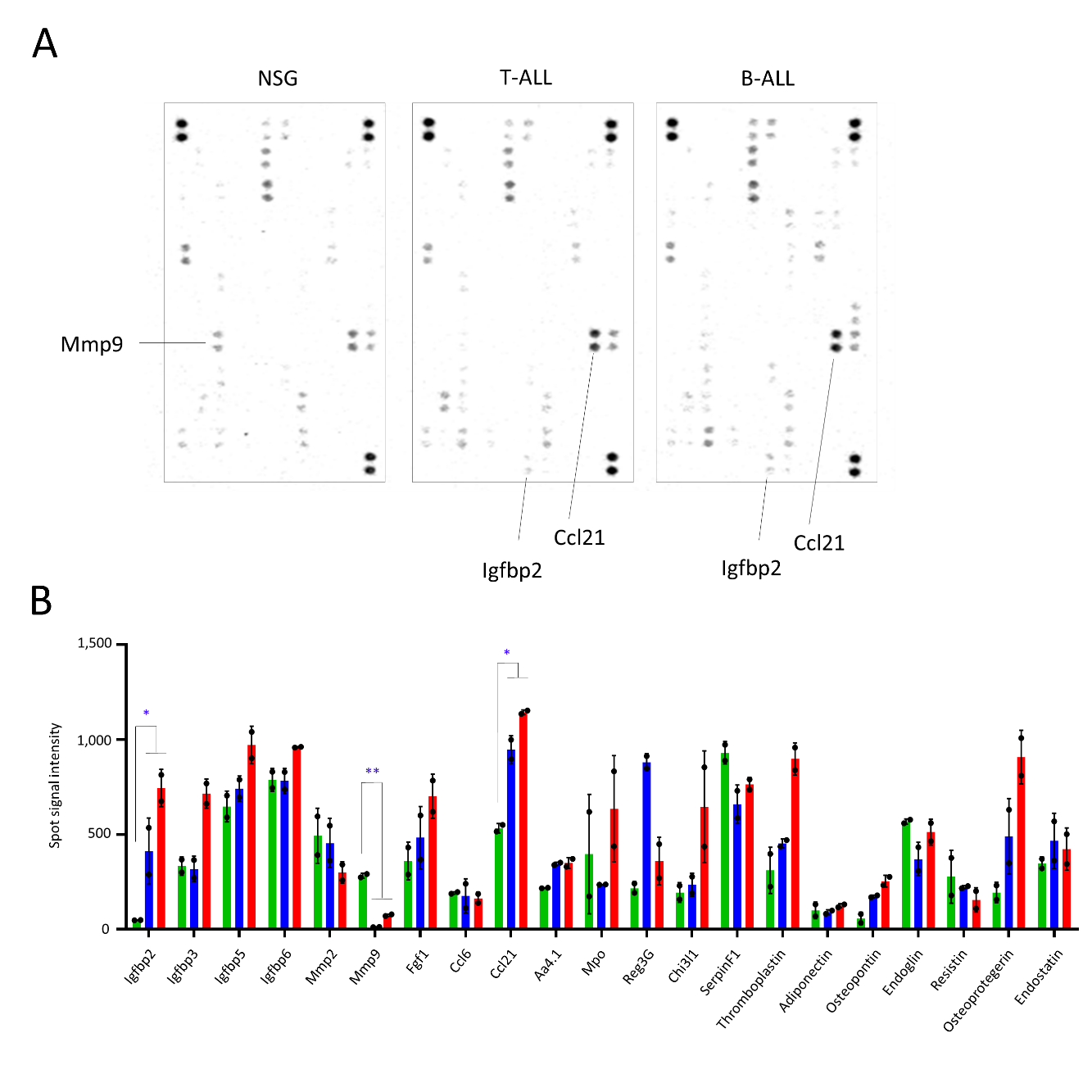
**

**Supplementary Fig. S9:** **The secretome is not affected in the BM niche of ALL PDX models.**

When mice, injected with 5×10^5^ T-ALL cells or 10^5^ B-ALL cells develop ALL disease (day 35), the secreted factors in the BM microenvironment are analyzed on a proteomic array. For each condition, the BM microenvironment of three mice are combined. **A** Pictures of the proteomic arrays. **B** Quantification showing that only three proteins (Igfbp2, Ccl21 and Mmp9) are dysregulated in ALL PDX models, compared with control NSG. Data are shown as mean ± SD; n=2 dots. P value measured by one-way Anova with Tukey’s multiple comparison test; *, P<0.05; **, P<0.01; no statistic is shown when P value is non-significant (P>0.05).

**
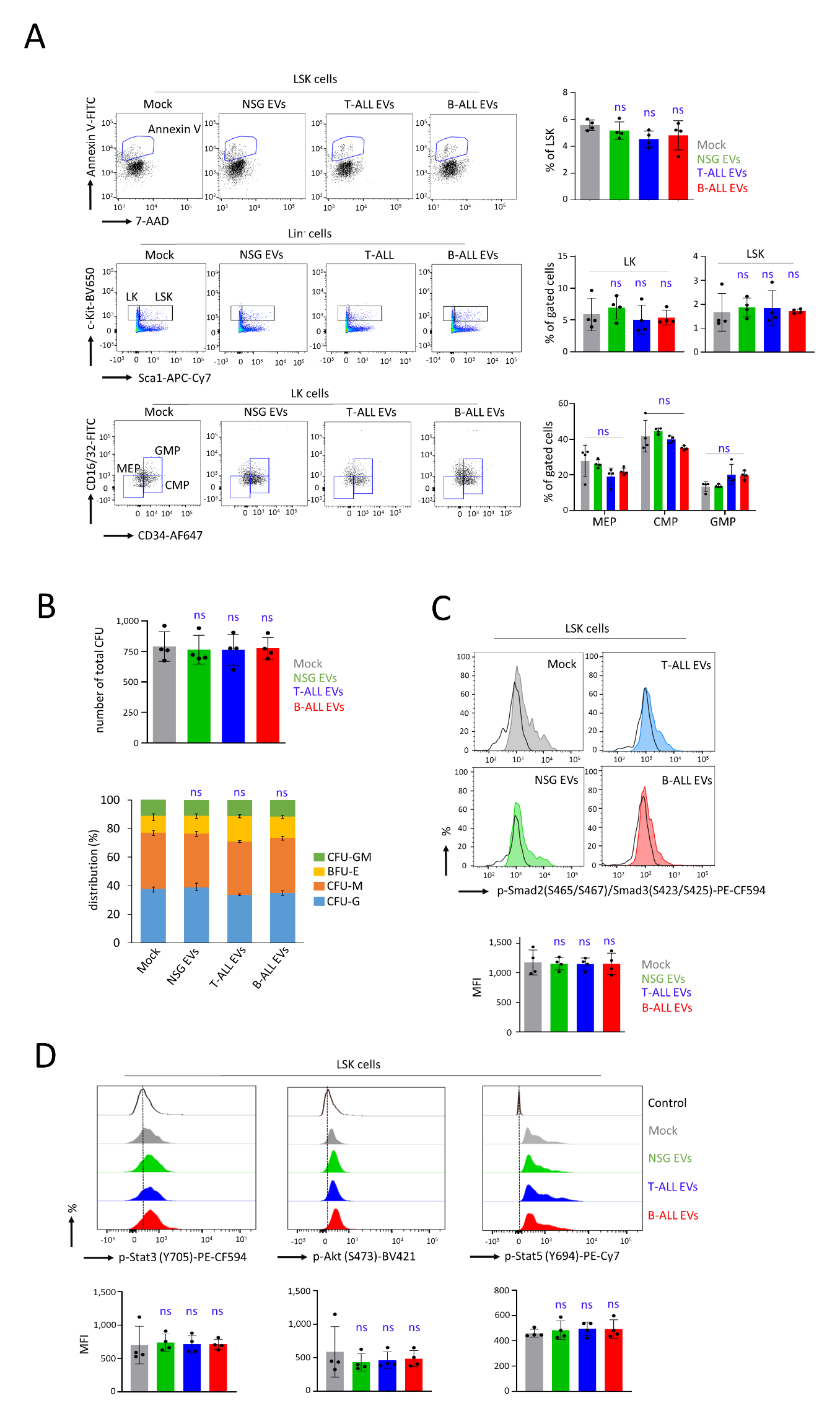
**

**Supplementary Fig. S10:** **ALL EVs do not alter the differentiation of HSPC.**

**A** Murine Lin^-^ cells are treated *ex vivo* with EVs (2×10^9^ particles) isolated from NSG control mice or ALL PDX mice. Apoptosis is assessed on LSK gating cells by flow cytometry, following Annexin V staining, 24 hours after an exposure with EVs. The percentage of early progenitors (LSK cells) show no variation *in vitro*, 24 hours after an exposure to ALL EVs, as assessed by flow cytometry. There is no impact on the distribution of mature progenitors such as mega-erythroid progenitor (MEP), common myeloid progenitor (CMP), granulocyte/macrophage progenitor (GMP), as assessed by flow cytometry and gating on LK (Lin^-^ c-Kit^+^) cells. **B** T-ALL and B-ALL EVs do not affect the growth of hematopoietic CFU *in vitro*. When Murine Sca1^+^ cells are treated *ex vivo* with T-ALL and B-ALL EVs, or NSG control EVs (2×10^9^ particles), there is no consequence on hematopoietic CFU colonies *in vitro*. After 24 hours of exposure to EVs, hematopoietic CFU is assessed on methylcellulose media. There is no impact of ALL EVs on the number, or the distribution of CFU colonies observed after 7 days of culture. **C** The TGFβ pathway is not affected on HSPC after an exposure to ALL EVs. Murine Lin^-^ cells are treated *ex vivo* with EVs (2×10^9^ particles) isolated from NSG control mice or ALL PDX mice. The percentage of phosphorylated Smad2/3 cells among LSK cells (HSPC) shows no variation *in vitro*, after an exposure to ALL EVs for 24 hours. **D** The phosphorylation of Stat3, Stat5 or Akt are not affected following an exposure to ALL EVs. Dashed lines corresponding to mean fluorescence intensity (MFI) for the control. On this figure, data are shown as mean ± SD; n=4 mice. P value measured by one-way Anova with Tukey’s multiple comparison test; ns, non-significant.

**
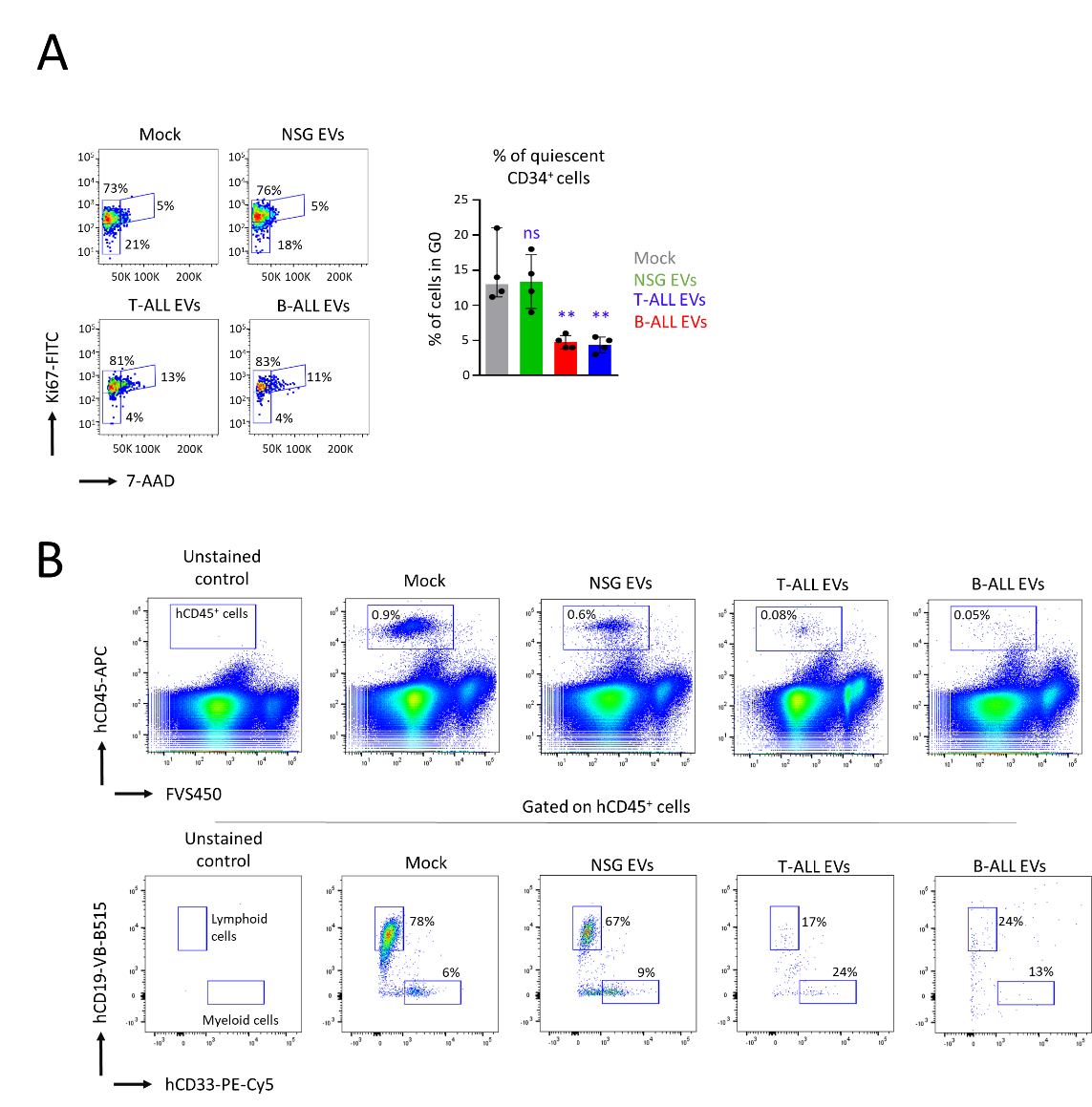
**

**Supplementary Fig. S11:** **ALL EVs isolated from PDX mice affect quiescence of CD34^+^ cord blood HSC.**

**A** Example of flow cytometry after Ki67 and 7-AAD staining on CD34^+^ cells isolated from cord blood and treated during 24 hours with EVs (2×10^9^ particles). Percentage of quiescent (G0) cells among CD34^+^ cells, following 24 hours of exposure with EVs. Data are shown as mean ± SD; n=4 cord blood. P value measured by one-way Anova with Tukey’s multiple comparison test; **, P<0.01; ns, non-significant. **B** Following 12 hours of exposure with EVs, CD34^+^ cells (10^5^ viable cells) are i.v. injected into sublethally irradiated recipient NSG mice. Four weeks after the transplantation, the human hematopoiesis reconstitution is assessed in BM, using human specific antibodies by flow cytometry. A reduced reconstitution is observed for CD34^+^ cells exposed to T-ALL and B-ALL EVs, while CD34^+^ cells untreated (Mock) or exposed to NSG EVs can reconstitute recipient NSG mice.


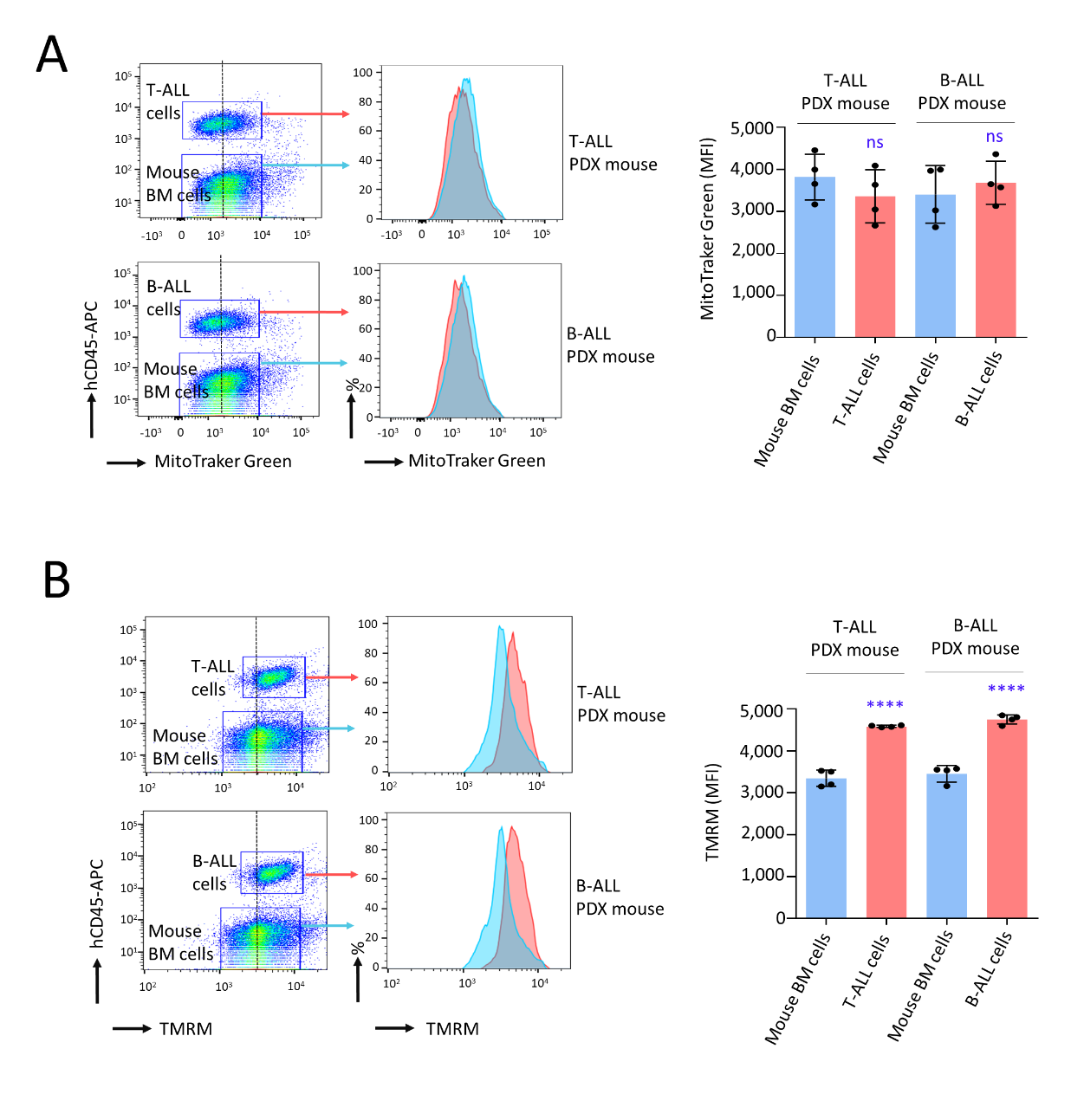


**Supplementary Fig. S12:** **ALL cells show the same quantity of mitochondria, but a relevant mitochondrial potential, compared with the endogenous control BM cells.**

**A** Quantification of the mitochondria measured by flow cytometry following MitoTraker staining. **B** The mitochondrial membrane potential measured by flow cytometry following TMRM staining. On this figure, data are shown as mean ± SD; n=4 mice. P value measured by one-way Anova with Tukey’s multiple comparison test; ****, P<0.0001; ns, non-significant. Dashed lines corresponding to mean fluorescence intensity (MFI) for mouse BM cells.


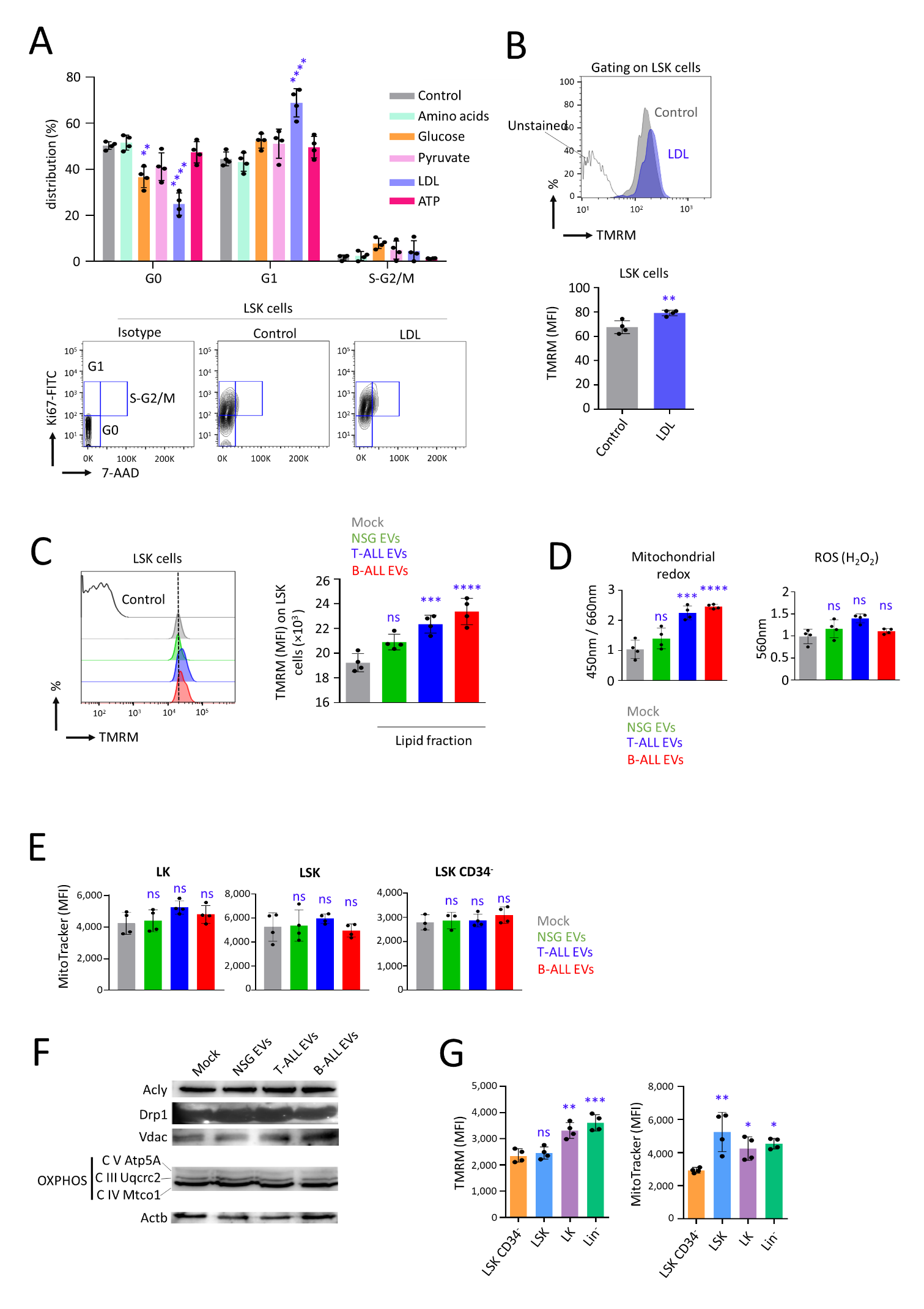


**Supplementary Fig. S13:** **ALL EVs enhance the mitochondrial potential of HSPC.**

**A** Lin^-^ cells are exposed *ex vivo* to different metabolites individually, and quiescence (% of cells in G0) is assessed on LSK cells by flow cytometry following 24 hours. Treatments with amino acids (1mM), glucose (1mM), pyruvate (1mM), ATP (1mM), as well as LDL (1mM). Data showing a loss of quiescence more particularly after treatment with LDL and glucose. **B** Flow cytometry after an exposure during 24 hours with LDL (1mM), showing the increased detection of the mitochondrial membrane potential states. Cells are treated with TMRM for 30min before flow cytometry recording. Data performed on Lin^-^ cells and gating on HSPC (LSK cells). Mean fluorescence intensity (MFI). Example of plot for TMRM recording is shown. Data are shown as mean ± SD; n=4 mice; P value measured by two-tailed Student’s unpaired t-test; **, P<0.01. **C** Flow cytometry data showing the increased detection of the mitochondrial membrane potential states following an exposure of Lin^-^ cells during 24 hours with the lipid fractions isolated from ALL EVs (2×10^9^ particles). Cells are treated with TMRM for 30min before flow cytometry recording. Data performed on Lin^-^ cells and gating on LSK cells. Example of plot for TMRM recording with dashed line corresponding to mean fluorescence intensity (MFI) for the Mock. **D** ALL EVs activate the mitochondrial activity in primitive hematopoietic cells. XTT assay (Absorbances at 450nm / 660nm) and Peroxidase assay (Absorbance at 560nm) used for the quantification of the mitochondrial oxidoreductase activity and the hydrogen peroxide reactive oxygen species (ROS), following 24 hours of treatment with EVs (2×10^9^ particles). Data are normalized to untreated controls (Mock); n=4 mice. **E** Flow cytometry showing that the mitochondrial labeling levels is not affected among LK, LSK and LSK CD34^-^ cells, following an exposure during 24 hours with EVs (2×10^9^ particles). Cells are treated with MitoTracker for 30min before flow cytometry recording. **F** Western blot on Lin^-^ cells exposed 24 hours with EVs (2×10^9^ particles) showing no impact on the expression of further proteins specifically involved in the metabolism in mitochondria. **G** Flow cytometry showing that the mitochondrial labeling levels (MitoTracker staining), as well as the mitochondrial potential (TMRM staining) are lower in HSC (LSK CD34^-^ cells) and HSPC (LSK cells), compared with LK and total Lin^-^ cells. Mean fluorescence intensity (MFI). On this figure, data are shown as mean ± SD; n=4 mice. P value measured by one-way Anova with Tukey’s multiple comparison test; *, P<0.05; **, P<0.01; ***, P<0.001; ****, P<0.0001; ns, non-significant. No statistic is shown when P value is non-significant (P>0.05).


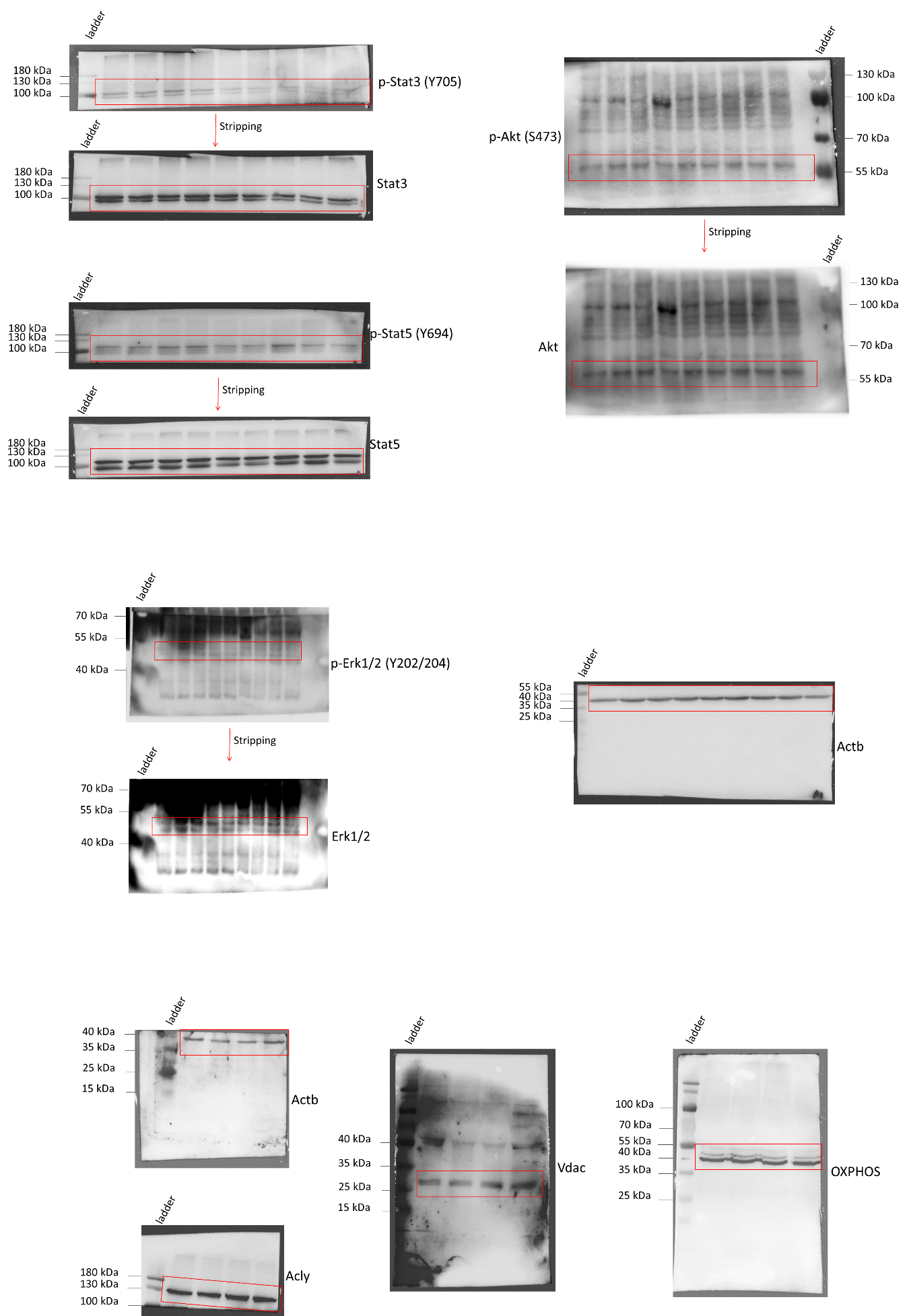


**Supplementary Fig. S14:** **Uncropped Western blots for Supplementary Fig. S6C and S13F.**

**Supplementary Table S1:** **The proteomic reveals many specific markers for large EVs.**

Compared with proteins already characterized in the literature (References). When a protein is specific for small size EVs (Exosomes) or large size EVs (Ectosomes), or specific for both, data showing the Score Mascot for NSG EVs, T-ALL EVs and B-ALL EVs is shown (grey). Twenty-three markers are specific to Ectosomes, while only one marker is specific to Exosomes.
