# Supplementary material for: Acute lymphoblastic leukemia-derived extracellular vesicles affect quiescence of hematopoietic stem and progenitor cells
Source: Cell Death Dis. 2022 Apr 12;13(4):337. doi: 10.1038/s41419-022-04761-5 (PMC9005650; doi:10.1038/s41419-022-04761-5)
Supplement: Supplementary file 2 — Supplementary Materials and Methods [file 41419_2022_4761_MOESM2_ESM.docx]

**Acute lymphoblastic leukemia-derived extracellular vesicles affect quiescence of hematopoietic stem and progenitor cells**

Aleksandra Georgievski,^1,2^ Anaïs Michel,^1^ Charles Thomas,^1,2^ Zandile Mlamla,^1,3^ Jean-Paul Pais de Barros,^1,2,3^ Stéphanie Lemaire-Ewing,^1,4^ Carmen Garrido^1,2,5^ and Ronan Quéré.^1,2,*^

^1^UMR1231, Inserm/Université Bourgogne Franche-Comté, Dijon, France.

^2^LipSTIC Labex, Dijon, France.

^3^Plateforme de Lipidomique Analytique, Université Bourgogne Franche-Comté, Dijon, France.

^4^Département de Biochimie, Hôpital Universitaire François Mitterrand, Dijon, France.

^5^Centre Georges François Leclerc-Unicancer, Dijon, France.

**Supplementary Materials and Methods**

**Supplementary References**

**Global lipidomic profiling on EVs**

Exosomes (10^10^ particles per sample) or PBS1× (200µl) were spiked with 1µl of Ultimate ONE SPLASH deuterated standards (Ref 330820L, Avanti Polar Lipids, Birmingham, USA). Total lipids were extracted according to the Folch method.^1^ The organic phase was collected, dried, and reconstituted in 100µl of CHCl3:MeOH (2:1) with 10mM Ammonium acetate. A pool of all sample extracts was used for quality control (QC). Briefly, the lipid extracts were analyzed on an Acquity BEH HILIC 1.7µm, 2,1x100mm column (Waters). Mobile phase A (acetonitrile/water (95/5 v/v) with 10mM Ammonium acetate, pH 8) and mobile phase B (acetonitrile/water (50/50 v/v) with 10mM Ammonium acetate) were used for lipid elution. The autosampler and column oven temperature were set at 15°C and 35°C, respectively. Ten microliters of each extract was injected in (+) positive and negative (-) ionization mode. A gradient at a flow rate of 0.5ml/min was as follows: 99.9-80% A in 10min; 2% A in 1min; 2% A for 2min; return to initial conditions, 2-99.9% A in 0.1min; and 99.9% A in 2.9min. Prior to each injection, the column was equilibrated for 10min at start conditions. The Exion LC system (Exion LC AD, AB Sciex), coupled on-line to the Triple Quadrupole-TOF 6600 mass spectrometer, equipped with DuoSpray electrospray ionization source (AB Sciex) was controlled via Analyst TF 1.8 software. Automatic calibrations via the calibrant delivery system were performed after every five injections. The instrument was operated at high resolution TOF MS and high sensitivity mode for Product ion scan with Sequential Window Acquisition of All Theoretical Mass Spectra (SWATH-MS 2.0) acquisition as the data independent approach. The SWATH MS1 and MS2 parameters were respectively set as follows: mass to charge (m/z) range, 50-1250 and 100-1200; accumulation time, 50ms and 20ms; collision energy, 10V, and 45V with a 15V collision energy spread. Additionally, Q1 isolation windows, 25Da; cycle time, 960ms, and RF transmission, m/z 40, 32.8%; m/z 130, 32.8%, and m/z 400, 34.3% were specified. The ion source parameters were set as follows: nebulizing gas, 50psi; heater gas, 60psi; curtain gas, 35 psi; declustering potential, 80 V; interface heater temperature, 500°C and ion spray voltage floating, 5500V (+), and 4500V (-). Data pre-processing was achieved with the MS-DIAL 4.60 software (<http://prime.psc.riken.jp/>). Briefly, raw data (.wiff format) were converted via the Analysis Base Files (ABF) converter (Reifycs, Tokyo, Japan).^2^ The (+) and (-) ESI acquired .abf files were analyzed separately. Peak detection parameters were set as follows: Retention time (Rt) range, 0.5-12 min; TOF-MS m/z range, 50-1250, and mass tolerance, 0.01 Da; SWATH MS2 m/z range, 100-1200, and mass tolerance 0.05; Smoothing method, linear weighted moving average; smoothing level, 3; minimum peak width, 3; minimum peak height, 500cps; and mass slice width, 0.1.^3,4^ Parameters for lipid assignment were as follows: Rt tolerance, 0.5min; mass tolerances, 0.01Da (MS1), and 0.05 Da (MS2); and an identification score cut-off of 80%.^5^ Peak alignment was based on the 2^nd^ QC sample, at a Rt of 0.1min and MS1 tolerance of 0.025Da. The Rt and MS1 factor were both set to 0.5, and gap filling by compulsory was applied. Rt correction was applied with at least one internal standard per lipid class at a Rt and mass tolerance of 0.5min and 0.0025Da, respectively. The alignment output (peak area data) from MSDIAL was used for downstream analyses. Molecular species without annotation and/or MS2 spectra, with non-conforming Rt, and coefficients of variance greater than 30% in QC samples were omitted. Peak areas of lipid species with the same molecular formula and Rt but different atomic arrangements were summed. For quantification, 17:0-18:1-d5 DG, PC, PE, PG, and PI; 17:0-d5 LPC, LPE, LPG, and LPI; as well as 16:0-17:1-16:0-d5 TG, d18:1-24:1-d7 Cer, and d18:1-16:1-d9 SM were used. The concentration of each detected lipid specie was determined from the ratio of the peak area of the given specie to the peak area of the corresponding internal standard. Except for PI, PG and their lysoforms, all lipid classes were quantified in (+) ESI mode. Lipid specie concentrations were expressed in nmoles per sample, and lipid class concentrations in molar percentage.

**UPLC proteomic analysis on EVs by LC-MS/MS**

Mass spectrometry was performed at the CLIPP facility (Dijon, France). EVs extracted from the BM of NSG mice (4 pooled mice) or ALL PDX models (2 pooled mice for T-ALL and 2 pooled mice for B-ALL) were reconstituted in 200µL of filtrated PBS1×. EVs were concentrated by ultrafiltration on a 3kDa filter (Thermo Fisher Scientific) at 14 000g for 15min. Lysis was obtained with SDS 2%, Tris 10mM and EDTA 1mM, and samples were concentrated to a volume of 50µL. Protein concentration was estimated (Detergent Compatible Dosage, BioRad), and 45µg was deposited on a 10%SDS-PAGE (Bio-Rad, Marnes-la-Coquette, France) at 150V for 15min. After Coomassie blue staining, gel bands were cut and proteins digested with 900ng of Trypsin overnight (in gel digestion). Peptides were extracted with acetonitrile 60% and formic acid 0.1% for one hour, followed by 10min with acetonitrile 100%. After dehydration (SpeedVac Vacuum, Thermo Fisher Scientific), peptides were purified under Microspin C18 columns (Harvard Apparatus, Holliston, USA) and reconstituted in 90µL of Acetonitrile 2% and formic acid 0.1%. Analysis was done by using a nanoUPLC (nanoRSLC, ThermoFisher) coupled with a mass spectrophotometer (Nano spray Advion TriVersa Nanomate and LTQ-Orbitrap Elite, Thermo Fisher Scientific). 3µL of sample was loaded on a column for enrichment (Acclaim PepMap C18, 75μm×20mm, Thermo Fisher Scientific) with solvent A (2% acetonitrile, 0.1% formic acid in water) at 5µL/min for 3min. Peptides were eluted by increasing concentration of solvent B (80% acetonitrile, 0.1% formic acid) from 2 to 25% over 160min at 300nL/min on a column (AcclaimPepMap, 75μm×250mm, 2μm, Thermo Fisher Scientific) maintained at 33°C. To identify proteins, we used the Mascot algorithm (v25.1, Mascot). We used databases for *Homo sapiens* and *Mus musculus* protein identification (Uniprot). Data from MS/MS were validated with ProlineStudio software (v2.0.1). The mass spectrometry proteomics data were deposited to the ProteomeXchange consortium via the PRIDE partner repository with the dataset identifier PXD021343. The deposited data were obtained from 45µg of proteins. Proteomic data were analyzed with the Multi Experiment Viewer (MEV, v4.9.0). Biological functions were obtained from the UniProt database. Principal Component Analysis (PCA) plots and Heatmap with clusters were made using the Clustvis software (https://biit.cs.ut.ee/clustvis/). To identified proteins specific for large size EVs (ectosomes) or small size EVs (exosomes), we used published datasets,^6-8^ as well as data from https://www.labome.com/method/Exosomes-Isolation-and-Characterization-Methods-and-Specific-Markers.html.

**UPLC amino acid analysis on EVs by LC-MS/MS**

EVs extracted from the BM of NSG mice or PDX models were reconstituted in 200µL of filtrated PBS1×. 50µL was used for the UPLC amino acid analysis performed using the AccQTag Ultra Derivatization Kit (186003836, Waters, Guyancourt, France). The UPLC column used was a UPLC C18 1.6μm 2.1×150mm (Waters). The UPLC used was a Waters Acquity UPLC system (Waters). We used solvent A (Formic acid 0.1% in water) and solvent B (Acetonitrile / 0.1% Formic acid) with a gradient of A/B (99:1) at 0.5mL/min for 13min at 5°C. We used the Amino acid standards solution (A6407, Merck) and the Amino acid standards physiological basics (A6282, Merck). Quality control used was the Recipe ClinChek Plasma Controls Level I and II (EEER10282, Waters). For the standard, we used the Metabolomics Amino Acid Mix Standard (MSK-A2-1.2, Cambridge Isotope Laboratories, Tewksbury, USA).

**CD34^+^ cord blood isolation, treatment with EVs and transplantation in NSG mice.**

Cord blood were obtained from the Etablissement Français du Sang (EFS, Bourgogne-Franche-Comté, France), under the reference number DECO-20-0014. Informed consent was obtained by EFS for all subjects. Cord blood mononuclear cells were isolated following Pancoll (Pan Biotech) density gradient centrifugation. CD34^+^ cells were recovered with magnetic beads (130-046-702, Miltenyi Biotec) on an AutoMACS Pro Separator (Miltenyi Biotec). CD34^+^ cells were cultured in 96-well U-bottom plates, with 200µL of the StemMACS media (Miltenyi Biotec), supplemented with PSA (Pan Biotech), human stem cell factor (SCF, 25ng/mL, 130-093-991, Miltenyi Biotec), human Interleukin 3 (IL3, 10ng/mL, 130-093-908, Miltenyi Biotec), human Interleukin 6 (IL6, 10ng/mL, 130-095-365, Miltenyi Biotec). Cells were divided in 4 wells (Mock, NSG EVs, T-ALL EVs, B-ALL EVs) and treated with 2×10^9^ particles. Twenty-four hours after the treatment, cells were analyzed by flow cytometry for Ki67 staining. CD34^+^ cells isolated from two cord blood samples were divided in 4 wells and treated with 2×10^9^ particles. Twelve-hours after the treatment, cells were i.v. injected (10^5^ viable cells) into NSG mice, irradiated 24-hours before the transplantation at sublethal dose of 3 Gray (BioMEP, Bretenière, France). To support the irradiation, 5×10^5^ support BM cells, isolated from a NSG mouse were also injected together with the CD34^+^ cells. After 4 weeks, engraftment was assessed by flow cytometry in BM.

**Culture of ALL cells and hematopoietic murine cells on MS5 feeder cells**

MS5 murine stromal feeder cells (ACC-441, DSMZ, Braunschweig, Germany) were cultured in 24-well plates with Iscove Modified Dulbecco Media (IMDM), 10% fetal bovine serum (Dominique Dutscher, Bernolsheim, France) and Penicillin-Streptomycin-Amphotericin (PSA, Pan Biotech). When the layer reached 80% confluence, we treated for three days with 2×10^9^ particles in serum-free media, then the media was changed and ALL cells were co-cultured for 24 hours on MS5 stromal feeder cells, in StemMACS media (Miltenyi Biotec) supplemented with PSA. After treatment, media was removed, cells binding on MS5 were trypsinized and the percentage of ALL cells was determined by flow cytometry and Hoechst staining. The same protocol was followed to study the binding of c-Kit^+^ murine cells and the percentage of progenitors and HSC were determined by flow cytometry and Hoechst staining. MS5 cells were also incubated for 24 hours with HSP70-ATTO488-stained EVs. Then the fluorescent EVs intake was analyzed by flow cytometry and fluorescence microscopy.

**MS5 cells migration assay**

Cell migration was assessed by wound healing assay. MS5 cells were grown in 6-well plates in complete media until 100% confluence. Cells were switched for 24 hours to serum-free media and a wound was created by scratching the center of the wells. Cultures were then cultured for three days in serum-free media supplemented in EVs isolated from the BM of control NSG mice or ALL PDX mice. Wounds were observed and images taken 24 and 72 hours after the scratching on a Cell Observer Zeiss (Zeiss) and analyzed with the WimScratch online tool (Wimasis, Ibidi, Gräfelfing, Germany).

**Western blots**

EVs were suspended in RIPA lysis buffer (150mM NaCl, 5mM EDTA (pH 8.0), 50mM Tris (pH 8.0), 1% NP-40, 0.5% sodium deoxycholate, and 0.1% SDS). On lysates, OD 620nm was measured to normalize the amount of the loaded sample. An appropriate quantity of protein was supplemented with 5× Laemmli buffer. Targeted proteins were separated on 10% SDS-PAGE gels and transferred to PVDF membranes. We used the proteome profiler antibody arrays (ARY028, Bio-Techne SAS, Noyal Châtillon sur Seiche, France) to detect mouse cytokines, chemokines and growth factors. For ALL PDX models and control NSG mice, we crushed BM in 2mL of PBS1× and removed cells by centrifugation (500g), then particles were removed from the supernatant by high-speed centrifugation (10 000g). The supernatant from three mice for each groups were pooled and we performed the proteome antibody arrays following the manufacturer’s instructions. To study effect of the EVs on MS5 signaling, we used anti-p-Stat3 (Y705) (1:1 000, #9145, Cell Signaling Technology, Ozyme, Saint-Cyr-l'Ecole, France), anti-Stat3 (1:1 000, #4904, Cell Signaling Technology), anti-p-Stat5 (Y694) (1:1 000, #9359, Cell Signaling Technology), anti-Stat5 (1:1 000, #94205, Cell Signaling Technology), anti-p-Akt (S473) (1:1 000, #4060, Cell Signaling Technology) and anti-Akt (1:1 000, #4691, Cell Signaling Technology), anti-p-Erk1/2 (Y202/204) (1:1 000, #4370, Cell Signaling Technology) and anti-Erk1/2 (1:1 000, #4695, Cell Signaling Technology). For Lin^-^ cells treated with EVs for 24 hours, we used anti-total OXPHOS antibody cocktail (1:1 000, ab110413, Abcam), anti-Drp1 (1:1 000, #8570, Cell Signaling Technology), anti-Vdac (1:1 000, #4661, Cell Signaling Technology), anti-Acly (sc-517267, Santa Cruz Biotechnology, Santa Cruz, USA). Anti-Actb (1:2 500, 612656, BD Biosciences) was used as a loading control. Appropriate secondary anti-mouse or anti-rabbit antibodies, conjugated with Horseradish Peroxidase were used (1:5 000, Cell Signaling Technology). Chemiluminescence was performed (Chemidoc, Bio-Rad), after applying ultra-sensitive enhanced chemiluminescent (ECL) substrate (SuperSignal West Femto Maximum Sensitivity, Thermo Fisher Scientific). Protein sizes were controlled by a protein ladder (Page Ruler Plus Prestained Protein Ladder, Thermo Fisher Scientific), and protein expression levels were assessed by using ImageJ (NIH Software, USA). Uncropped Western blots are shown in Supplementary **Fig. S14**.

**Transmission electron microscopy**

For transmission electron microscopy (TEM), EVs morphology was observed by negative staining. Twenty microliters of EVs sample were applied to glow-discharged, carbon-coated collodion 200-mesh Ni grids and incubated for 5-10min, at 4°C. Grids were then stained using 2% UA (pH 7) in water for 5-15min. Visualizations were performed using a TEM (HITACHI HT7800) operating at 100kV and equipped with AMT cameras (AMT, Woburn, USA).

**Artificial proteoliposomes with HSP70 expression**

L-α-Phosphatidylcholine (P7443, Merck) and Cholesterol (C8667, Merck) were weighted and dissolved in chloroform, at a ratio of 16:3 (w/w). Using a nitrogen stream, we evaporated the chloroform in order to obtain a lipid film. The latter was then hydrated in filtered PBS1× (0.1µm) at 65°C for 30min before undergoing 5 cycles of 30s on/off ultrasound at amplitude 20%. Liposomes were then incubated a second time at 65°C for 30min. In order to include HSP70, 0.3% triton X100 (Merck) and 10ng or 20ng of the recombinant HSP70 (ADI-NSP-555, Enzo Life Sciences, Villeurbanne, France) were incubated with the liposomes at room temperature and under agitation for 45min. A control was performed without HSP70 inclusion. Once HSP70 was included, the proteoliposomes were incubated with HSP70 peptide aptamer conjugated with the ATTO488, at 4°C for 30min before undergoing two dialysis at 4°C in a cold room; one of 2 hours and the second overnight, both in one liter of PBS1×. After dialysis, proteoliposomes sizes and concentrations were determined by NTA. We furthermore checked for presence of HSP70 by flow cytometry (ATTO488 expression). Lin^-^ cells, isolated from NSG mice, cultured in 96-well U-bottom plates in 200µL of PBS1×, were treated for 2 hours with 2×10^9^ proteoliposomes. Binding of HSP70 proteoliposomes was then assessed on Lin^-^ cells by flow cytometry.

**Supplementary References**

1 Folch, J., Lees, M. & Sloane Stanley, G. H. A simple method for the isolation and purification of total lipides from animal tissues. *J Biol Chem* **226**, 497-509 (1957).

2 Tsugawa, H. *et al.* MS-DIAL: data-independent MS/MS deconvolution for comprehensive metabolome analysis. *Nat Methods* **12**, 523-526, doi:10.1038/nmeth.3393 (2015).

3 Drotleff, B., Illison, J., Schlotterbeck, J., Lukowski, R. & Lammerhofer, M. Comprehensive lipidomics of mouse plasma using class-specific surrogate calibrants and SWATH acquisition for large-scale lipid quantification in untargeted analysis. *Anal Chim Acta* **1086**, 90-102, doi:10.1016/j.aca.2019.08.030 (2019).

4 Tsugawa, H. *et al.* A lipidome atlas in MS-DIAL 4. *Nat Biotechnol* **38**, 1159-1163, doi:10.1038/s41587-020-0531-2 (2020).

5 Kind, T. *et al.* LipidBlast in silico tandem mass spectrometry database for lipid identification. *Nat Methods* **10**, 755-758, doi:10.1038/nmeth.2551 (2013).

6 Keerthikumar, S. *et al.* Proteogenomic analysis reveals exosomes are more oncogenic than ectosomes. *Oncotarget* **6**, 15375-15396, doi:10.18632/oncotarget.3801 (2015).

7 Kowal, J. *et al.* Proteomic comparison defines novel markers to characterize heterogeneous populations of extracellular vesicle subtypes. *Proc Natl Acad Sci U S A* **113**, E968-977, doi:10.1073/pnas.1521230113 (2016).

8 Surman, M., Stepien, E., Hoja-Lukowicz, D. & Przybylo, M. Deciphering the role of ectosomes in cancer development and progression: focus on the proteome. *Clin Exp Metastasis* **34**, 273-289, doi:10.1007/s10585-017-9844-z (2017).
